# A 12-Connected [Y_4_((*μ*_3_-OH)_4_]^8+^ Cluster-Based Luminescent Metal-Organic Framework for Selective Turn-on Detection of F^−^ in H_2_O

**DOI:** 10.3390/molecules28041893

**Published:** 2023-02-16

**Authors:** Juan Li, Airong Wang, Shiming Qiu, Xiaoli Wang, Jiaming Li

**Affiliations:** 1 Guangxi Key Laboratory of Green Chemical Materials and Safety Technology, College of Petroleum and Chemical Engineering, Beibu Gulf University, Qinzhou 535011, China; 2 College of Chemistry and Biological Engineering, Guangxi Minzu Normal University, Chongzuo 532200, China

**Keywords:** cluster-based metal-organic framework (MOFs), yttrium, 3-nitro-4,4′-biphenyldicarboxylic acid, synthesis, fluorescence, crystal structure

## Abstract

Fluoride ion (F^−^) is one of the most hazardous elements in potable water. Over intake of F^−^ can give rise to dental fluorosis, kidney failure, or DNA damage. As a result, developing affordable, equipment-free and credible approaches for F^−^ detection is an important task. In this work, a new three dimensional rare earth cluster-based metal-organic framework assembled from lanthanide Y(III) ion, and a linear multifunctional ligand 3-nitro-4,4′-biphenyldicarboxylic acid, formulated as {[Y(*μ*_3_-OH)]_4_[Y(*μ*_3_-OH)(*μ*_2_-H_2_O)_0.25_(H_2_O)_0.5_]_4_[*μ*_4_-nba]_8_}*_n_* (**1**), where H_2_nba = 3-nitro-4,4′-biphenyldicarboxylic acid, has been hydrothermally synthesized and characterized through infrared spectroscopy (IR), elemental and thermal analysis (EA), power X-ray diffraction (PXRD), and single-crystal X-ray diffraction (SCXRD) analyses. X-ray diffraction structural analysis revealed that **1** crystallizes in tetragonal system with *P*$\overset{¯}{4}$2_1_*m* space group, and features a 3D framework with 1D square 18.07(3)^2^ Å^2^ channels running along the [0,0,1] or *c*-axis direction. The structure of **1** is built up of unusual eight-membered rings formed by two types of {Y_4_O_4_} clusters connected to each other via 12 *μ*_4_-nba^2−^ and 4 *μ*_3_-OH^−^ ligands. Three crystallographic independent Y^3+^ ions display two coordinated configurations with a seven-coordinated distorted monocapped trigonal-prism (YO_7_) and an eight-coordinated approximately bicapped trigonal-prism (YO_8_). **1** is further stabilized through O-H⋯O, O-H⋯N, C-H⋯O, and π⋯π interactions. Topologically, MOF **1** can be simplified as a 12-connected 2-nodal *Au_4_Ho* topology with a Schläfli symbol {4^20^·6^28^·8^18^}{4^3^}_4_ or a 6-connected uninodal *pcu* topology with a Schläfli symbol {4^12^·6^3^}. The fluorescent sensing application of **1** was investigated to cations and anions in H_2_O. **1** exhibits good luminescence probing turn-on recognition ability toward F^−^ and with a limit detection concentration of F^−^ down to 14.2 μM in aqueous solution (*K*_ec_ = 11403 M^−1^, *R*^2^ = 0.99289, *σ* = 0.0539). The findings here provide a feasible detection platform of LnMOFs for highly sensitive discrimination of F^−^ in aqueous media.

## 1. Introduction

The design and the synthesis of macropore open metal-organic frameworks (MOFs) or coordination polymers (CPs) based on metal centres or clusters are of great interest for their promising potential applications in several different fields as for example in gas storage and separation, host–guest chemistry, catalysis, and fluorescence sensing [1,2,3,4,5,6,7,8]. Current interest is mainly devoted to the construction of metal–organic frameworks (MOFs) or coordination polymers (CPs) through coordination of metal ions with multifunctional organic ligands as connectors [9,10,11,12,13,14,15]. LnMOFs (MOFs based on trivalent lanthanides) containing dicarboxylate linkers show an intriguing variety of crystal structures and topologies due to their usually high coordination number leading to cluster units and polymeric chains most frequently observed for these compounds. In particular LnMOFs, in which both Ln^3+^ and linkers can be used to give rise to luminescence materials (through antenna effect) with increased brightness and emission quantum yield, are a very promising class of materials in a wide range of optical applications including lasers, imaging agents, lighting systems, display applications, and ions recognition [16,17,18,19,20]. Especially, polynuclear hydroxidolanthanide based cluster metal-organic frameworks are important type of LnMOFs and attracting increasing attentions in view of their optical, electronic and catalytic properties, which may primarily lead to potential various applications such as optoelectronic devices, biocatalyst and luminescent materials [21]. Multifarious polynuclear hydroxidolanthanide clusters have been reported in the past two decades [22]. The reported structures show that the basic structural skeletons are classified into three motifs, i.e., square pyramids and cubane-like clusters with the [Ln_4_(μ_3_-OH)_4_]^8+^ building block, and chair-like cores with the [Ln_6_(μ_3_-OH)_4_]^14+^ building unit [23,24]. Commonly, the tetranuclear hydroxidolanthanide clusters belong to a cubane-like building unit [25] and two cubanelike entities can be combined to a heptanuclear [Ln_7_(μ_3_-OH)_8_]^13+^ cluster sharing one vertex [26]. It is worth mentioning that four or five vertex sharing cubane-like cluster units centering around a halide anion can be assembled to dodeca- and pentadecanuclear clusters [27] with wheel structures. The pentanuclear square-pyramid unit appears to be another common structural motif in hydroxidolanthanide clusters [28]. The [Eu_5_(μ_4_-OH)(μ_3_-OH)_4_]^10+^ cluster has been reported [29] and the square-pyramidal Ln_5_ unit can be considered as a building block for hexa- [30], nona- [31] and tetradecanuclear [32] clusters sharing their apexes or the square bases. To the best of our knowledge, the majority of hexanuclear [Ln_6_(μ_6_-O)(μ_3_-OH)_8_]^8+^ skeletons are octahedral while the [Ln_6_(μ_3_-OH)_4_]^14+^ chair-like core as building block has rarely been reported. Most of the reported works devoted to polynuclear hydroxidolanthanide MOFs focus on the choice of the Ln^3+^ or Ln-clusters. Actually, although the validity of the approach has been demonstrated for rare earth ions, there are only a few examples of hydroxidolanthanide coordination polymers in which polynuclear complexes act as metallic centers.

Chen and his collaborators reported a series of Y(III)-based clusters, Y_12_, Y_34_, and Y_60_, containing 4, 12, and 24 cubane-like [Y_4_(μ_3_-OH)_4_]^8+^ units, respectively, was realized by using suitable organic ligands and controlling the acidity of the hydrolysis reaction [33]. The formulated as [Y_4_(OH)_4_(25p)_4_(H_2_O)_3_].H_2_O and [Y_6_(OH)_8_(25p).5(H_2_O)_2_] (25p = 2,5-pyridinedicaboxylate) lanthanide–organic frameworks, have been obtained by Abdelbaky and his coauthors as single phases under hydrothermal conditions. While the structure of [Y_6_(OH)_8_(25p).5(H_2_O)_2_] is based on two independent cuban-like[Y_4_(OH)_4_]^8+^ clusters, which are joined together through Y1 cation leading to the formation of hexanuclear[Y_6_(OH)_8_]^10+^ clusters, which in turn are joined via Y2 cation resulting in infinite inorganic chain, and each chain is interconnected to six adjacent chains through 2,5-pyridinedicaboxylate ligands leading finally to 3D framework [34]. Interestingly, Liu et al. controlled hydrolysis of complexes Cp_2_Ln(η^2^-HBzimt)(THF)_2_, CpLn(η^2^-HBzimt)_2_(THF) and [(Cp_2_Ln)(THF)]_2_(μ-Bzimt) resulting in the same tetranuclear complexes [CpLn(μ_3_-OH)( μ-η^1^: η^2^-HBzimt)]_4_ (Ln = Yb, Er, Y), indicating that the hydrolysis selectivity greatly depends on the number of coordinated cyclopentadienyl groups [35]. In addition, Mishra et al. reported a partially hydrolyzed [Y_4_(μ_3_-OH)_4_(μ-η^1^:η^1^-TFA)_6_(η^1^-TFA)(η^2^-TFA)(THF)_3_(DMSO)(H_2_O)].6THF (TFA = trifluoroacetate), which was obtained with moderate yield, by crystallization of thermally treated batches of Y(TFA)_3_(H_2_O)_3_ from THF/DMSO at room temperature [36]. Despite all this, however, those containing [Y_4_(μ_3_-OH)_4_]^8+^ clusters LnMOFs are still scare compared with other rare earth polynuclear hydroxidolanthanide based cluster metal-organic frameworks.

Similar to the transition metal cluster based MOFs self-assembly, the choice of ligand is crucial in the formation of lanthanide functional coordination material. Because lanthanide ions can be considered as hard acids according to Pearson’s theory [37], the choice of the ligand is of first importance as far as polynuclear hydroxidolanthanide based cluster metal-organic frameworks is concerned. Most of them are built with the classical benzoic multicarboxylate ligands, such as terephthalate [16,38,39], 2-hydroxyterephthalic [18], 2,5-dihydroxyterephthalic, 1,2,3- or 1,3,5-benzenetricarboxylic, 1,2,4,5-benzenetetracarboxylic and 4,4′-biphenyldicarboxylate acid, which are versatile building blocks to obtain various high dimensional lanthanide frameworks due to their variety of bridging abilities [40,41,42,43,44,45,46,47,48,49,50,51,52,53,54,55,56,57,58,59,60].

**Scheme 1:**
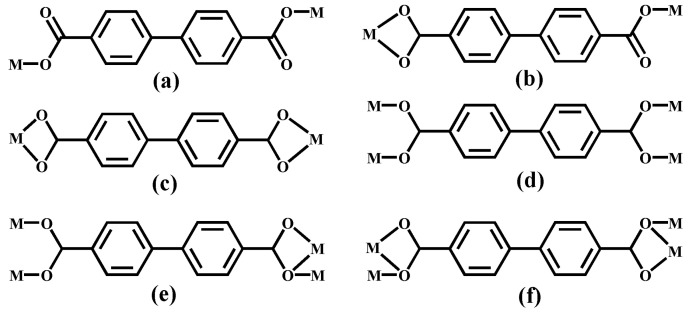
(**a**–**f**) The familiar 6 coordination modes of 4,4′-biphenyldicarboxylate ligand.

4,4′-biphenyldicarboxylate acid (H_2_bda) is an excellent candidate (Scheme 1) for assembling novel CPs/MOFs by incorporating appropriate metal ions in different forms which can lead to products possessing beautiful and interesting topological structures, ranging from chains and sheets to three-dimensional (3D) porous structures [61,62,63]. H_2_bda ligand has been widely used to synthesize high dimensional lanthanide frameworks, various supramolecular structures with fascinating properties displayed. On the other side, as the most relevant derivatives of H_2_bda, such as 3-nitro-4,4′-biphenyldicarboxylic [64], 3-amino-4,4′-biphenyldicarboxylic [65], 3,3′-dinitro-4,4′-biphenyldicarboxylic [66,67], and 3,3′-diamino-4,4′-biphenyldicarboxylic acid [68,69], have hitherto remained largely unexplored in the field of CPs/MOFs compared with the well-studied ligands H_2_bda. This article takes 3-nitro-4,4′-biphenyldicarboxylic (abbreviated as H_2_nba) as an example to elaborate the potential advantages of these ligands for constructing novel MOFs. H_2_nba features two biphenyl rings and a nitro group in the 3-position. H_2_nba was selected as a ligand, which has the following characteristics: (i) The two carboxyl groups has four donor atoms with the dihedral angle 180°, are able to establish bridges between several metal nodes through variety of coordination modes and conjugated aromatic ring allows it to become a rigid linker. (ii) Nitro group in H_2_nba provides more hydrogen bonding donor or acceptor sites for supramolecular interactions. In particular, the two nitro O donors play a key role in the formation of potential hydrogen bonded interactions. (iii) Ditopic linear rod-like topology introduces low steric hindrance, thus, allowing formation of porous and stabilized framework. (iv) Biphenyl ring functions as a big space mediator for transmitting exchange interaction between metal centres. (v) Moreover, the two aromatic rings of H_2_nba may take part in π–π and C–H⋯π stacking interactions. Despite numerous MOFs or CPs have been synthesized based on 4,4′-biphenyldicarboxylate acid, little attention have been focused on its derivatives, such as H_2_nba. To the best of our knowledge and in a search of the Cambridge Crystallographic Database (CSD), however, there is only one crystal structure report of H_2_nba as ligand for the construction of MOFs or CPs by our research group to date [64].

Recently, the development of fluorescence MOFs detection/capture material to recognize biologically and environmentally excess F^−^ ions has attracted great attention because of their indispensable roles in physiological processes [70,71,72]. Low concentrations of F^−^ have beneficial effect on dental health and the clinical treatment of osteoporosis [73,74]. However, excess intake of F^−^ can lead to dental fluorosis, kidney failure, or DNA damage [75,76]. The World Health Organization recommends F^−^ contents in drinking water of less than 1.5 ppm, and overexposure is known to cause dental fluorosis, osteoporosis, and other serious health problems [71]. Therefore, monitoring fluoride concentration on site or in field is very important for public health. Traditional methods including ion chromatography and ion-selective electrode are commonly used for F^−^ detection [77,78]. However, complicated procedures, high cost, or low mobility can prevent their application for on-site monitoring. Therefore, new solution for screening F^−^ pollution aims to develop equipment-free and inexpensive methods for non-expert users, especially in remote rural areas [79,80]. The Ln-MOFs materials with open metal sites is very useful to construct anion receptors based on anion-metal interaction, or hydrogen bonding interaction. Among various anions, the luminescent turn-on detection of pollutional anions, such as F^−^, is the most urgent need for increasing water contamination and environmental problems closely related with our living.

In order to completely fulfill the several important factors mentioned above and ongoing our research work in this area [81,82,83,84,85], we used H_2_nba as organic ligand for [Y_4_(OH)_4_]^8+^ cluster center. A Y(III)-based MOF, {[Y(*μ*_3_-OH)]_4_[Y(*μ*_3_-OH)(*μ*_2_-H_2_O)_0.25_(H_2_O)_0.5_]_4_[*μ*_4_-nba]_8_}*_n_* (**1**) has been successfully synthesized by hydrothermal methods and characterized by infrared spectrum (IR), elemental analysis (EA), power X-ray diffraction (PXRD), single-crystal X-ray diffraction (SCXRD) and thermogravimetry (TG). In addition, the liquid state luminescence properties of **1**, and the fluorescent detection of cations/anions of **1** were investigated at room temperature in detail.

## 2. Results and Discussion

### 2.1. Description of the Structure of {[Y(μ_3_-OH)]_4_[Y(μ_3_-OH)(μ_2_-H_2_O)_0.25_(H_2_O)_0.5_]_4_[μ_4_-nba]_8_}_n_ (**1**)

**Figure 1:**
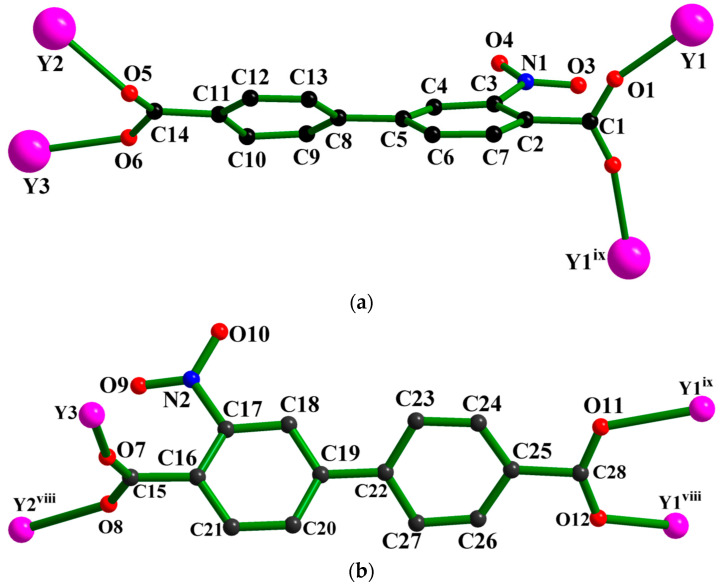
(**a**,**b**) The coordination mode of two crystallographically independent nba^2−^ ligands in **1**.

**Figure 2:**
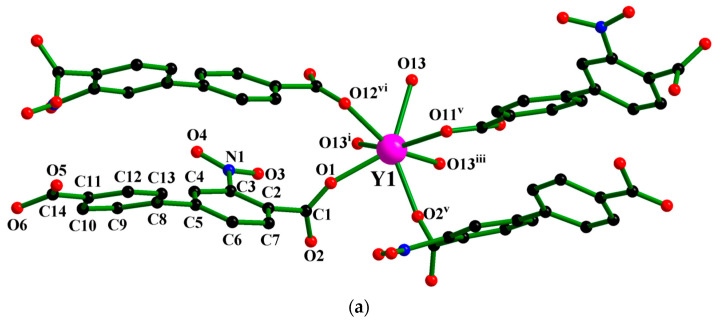

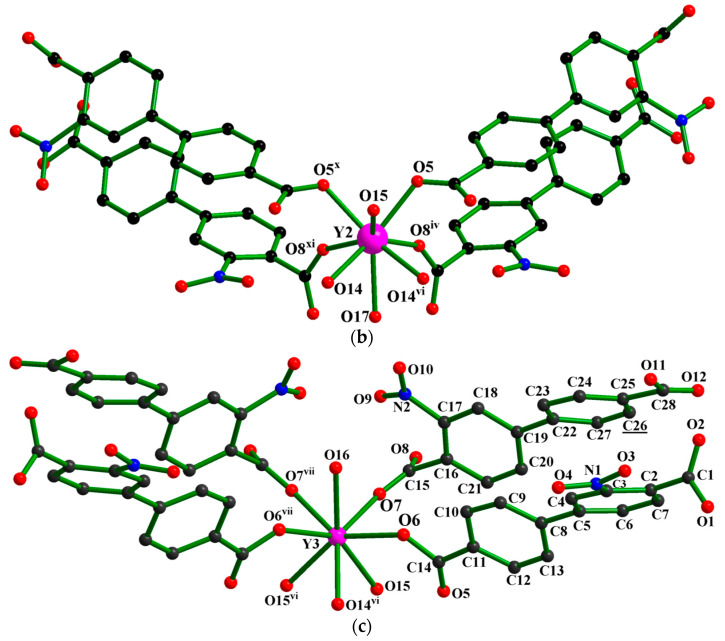
(**a**) The coordination environment for Y1 in **1**; (**b**) The coordination environment for Y2 in **1**; (**c**) The coordination environment for Y3 in **1**.

X-ray single-crystal diffraction analysis revealed that the crystal structure of **1** is composed of a 3D neutral metal–organic framework based on two asymmetrical, irregular tetranuclear yttrium cubes {Y_4_O_4_} connected to twelve individual nba^2−^ ligands, in a tetragonal system with a *P*$\overset{¯}{4}$2_1_*m* space group (Table S1 from Supplementary Materials). The asymmetric unit of **1** contains a quarter of the formula as {[Y(μ_3_-OH)][Y(μ_3_-OH)(μ_2_-H_2_O)_0.25_(H_2_O)_0.5_][L]_2_}_4_, i.e., one full and two halves-occupied Y^3+^ cations, two nba^2−^ anions, one full and two halves-occupied OH^−^ anions, as well as a half and a quarter-occupied coordinated H_2_O (Figure S1 from Supplementary Materials). As shown in Figure 1, the two crystallographically independent nba^2−^ ligands adopt the same coordination mode linking four Y^3+^ ions in a *μ*_4_-*η*^1^:*η*^1^:*η*^1^:*η*^1^ monodentate fashion, according to the bond characteristics of carboxyl units. Three crystallographic independent Y^3+^ ions can be classified into two groups based on their coordination numbers. For Y1 ion fully occupied at 8f site, it is coordinated by seven O atoms from four *μ*_4_-*η*^1^:*η*^1^:*η*^1^:*η*^1^ monodentate bridging crystallographically nonequivalent carboxyl radicals (O1, O2^v^, O11^v^, O12^vi^) and three hydroxyl groups from three symmetry-related *μ*_3_-OH^−^ ligands (O13, O13^i^, O13^iii^) (Figure 2a). Its coordination polyhedron can be described as a seven-coordinated distorted monocapped trigonal-prism (Figure S2a from Supplementary Materials).This symmetry is in good agreement with what had been encountered in the crystal structure of the anhydrous complex [21]. While both Y2 and Y3 ions located at 4e site with a site occupancy factor of 0.50, they are coordinated by eight O atoms from two pairs imposed symmetry bridging carboxyl radicals (O5, O5^x^, O8^iv^, and O8^xi^ for Y2; O6, O6^vii^, O7, and O7^vii^ for Y3) and three hydroxyl groups from a pair of symmetry-related *μ*_3_-OH^−^ and an isolated *μ*_3_-OH^−^ ligands (O14, O14^vi^, and O15^vi^ for Y2; O14^vi^, O15, and O15^vi^ for Y3), and the last one from the coordinated water molecule (O17 for Y2; O16 for Y3) (Figure 2b,c). Their coordination geometries may be described as an approximately bicapped trigonal-prism (Figure S2b from Supplementary Materials). More detailed coordination information of Y^3+^ ions is summarized and provided in Table S2 (from Supplementary Materials). The eight oxygen atoms coordinated to Y3 as similar as Y2 except that O16 (O16 located at 8f site with a site occupancy factor of 0.50) monodentate bonded to Y3 instead of *μ*_2_-bridging coordination fashion O17 atom (O17 located at 2c site with a site occupancy factor of 0.25). The carbonate radical exhibits a *μ*_4_-*η*^1^:*η*^1^:*η*^1^:*η*^1^ mode to bond to four Y^3+^ ions (Figure 1), but the carboxylic acid on the benzene ring containing the nitro group uses an *anti*-coordination mode to coordinate with the Y atoms (Y1, Y1^ix^, Y2^viii^, and Y3), while the carboxylate on the benzene ring without nitro group adopt a *cis*-coordination fashion to coordinate with Y ions (Y2, Y3, Y1^viii^, and Y1^ix^) substituted for an anti-coordination mode. All hydroxyl groups apply the same *μ*_3_-mode to coordinate with Y^3+^ ions as reported in the literature [21,22,26,29,31,40,65]. The Y–O bonds have average distances of 2.262(13) Å and the O–Y–O bond angles are in the range of 63.2(8)–150.3(6)°, all in the normal parameters comparison to the related Y-MOFs or Y-CPs [64,65,66,67,68,69].

**Figure 3:**
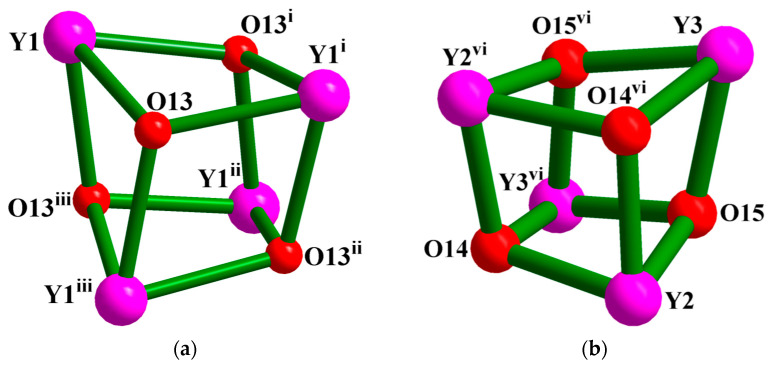
(**a**) The Y1-containg quadnuclear {Y_4_O_4_} cluster secondary building unit; (**b**) The Y2-Y3-containg quadnuclear {Y_4_O_4_} cluster secondary building unit.

Four adjacent Y(III) ions are respectively bridged by four hydroxyl groups in a *μ*_3_-OH^−^ bridging fashion to give two asymmetrical quadnuclear {Y_4_O_4_} cluster secondary building unit (SBU) (Figure 3a,b), namely, Y1-containg (denoted by symbol A as follows) and Y2-Y3-containing clusters (denoted by symbol B as follows). Every three adjacent Y^III^ ions are linked by one μ_3_-OH group in the centre, which is slightly above the basal face and the Y–O (μ_3_-OH) distances range from 2.32(9) to 2.45(8) Å. Both tetranuclear {Y_4_O_4_} entities can be described as a severe distorted cube with four Y(III) ions and four hydroxyl O atoms localized on eight vertexes, while the {Y_4_} or {O_4_} constitutes a perfect tetrahedron (Figure S3a–g from Supplementary Materials). In addition, four Y^3+^ ions of each cube is capped by four symmetry-related *μ*_3_-hydroxyde groups for A, and with two pairs of imposed symmetry *μ*_3_-OH^−^ involved in B. In the {Y_4_O_4_} cluster, the average Y⋯Y separation is 3.74(8) Å, which is slightly larger than the sum of metal radii (3.62 Å) of Y(III) centers, implying obvious cuprophilic interactions. While the average O⋯O distance is 2.87(7) Å, which is slightly more than the sum of van der Waals radii (2.8 Å) of O centers, implying obvious O⋯O hydrogen bonding interactions.

**Figure 4:**
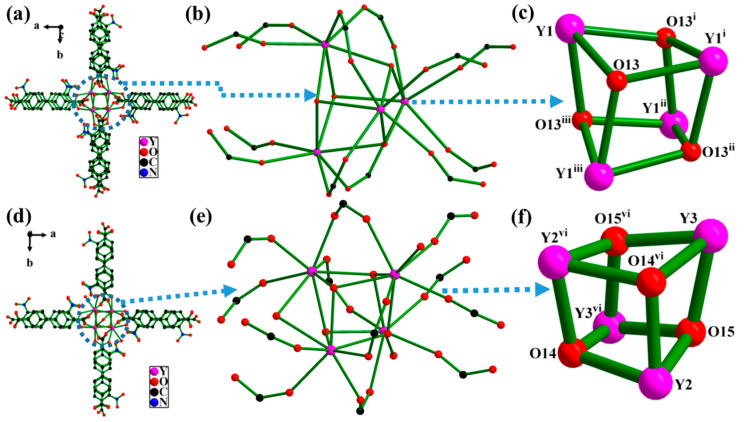
(**a**) and (**b**) The Y1-containing {Y_4_O_4_} cluster linked by 12 nba^2−^ ligands; (**c**) The Y1-containing {Y_4_O_4_} cluster; (**d**) and (**e**) The Y2/Y3-containing {Y_4_O_4_} cluster linked by 12 nba^2−^ ligands; (**f**) The Y2/Y3-containing {Y_4_O_4_} cluster.

**Figure 5:**
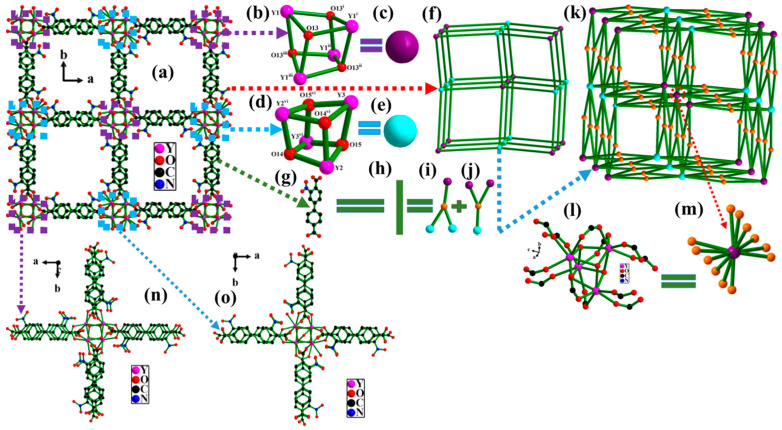
(**a**–**o**). The simplified schematic diagram of 6-connected *pcu* net with a Schläfli symbol {4^12^·6^3^} or 12-connected *Au_4_Ho* topology with a point (Schläfli) symbol {4^20^·6^28^·8^18^}{4^3^}_4_.

Interestingly, all {Y_4_O_4_} clusters can be joined by eight monodentate {Y_4_O_4_} manner carboxyl groups (Figure 4) along *a*- and *b*-axis to form an infinite two-dimensional (2-D) organic-inorganic lattice with a −ABAB− mode of a square dimension of 18.07(3) × 18.07(3) Å^2^ (the center separation of A and B). Furthermore, these 2-D nets are linked to four bidentate {Y_4_O_4_} manner carboxylate radicals and stacked to each other with a −AAAA− or a −BBBB− style of a layer thickness of 7.94(3) Å (the center separations of A-A and B-B), leading to a three-dimensional (3-D) metal-organic framework with a one-dimensional (1-D) 18.07(3) × 18.07(3) Å^2^ (the center distances between the A and B) square channel along the *c*-axis or [0,0,1] direction (Figure 5 and Figure S4 from Supplementary Materials).

Topologically, if each {Y_4_O_4_} core serves as a 12-connected node and is linked by 12 nba^2−^ ligands, and each nba^2−^ chelates or bridges three {Y_4_O_4_} clusters acting as a 3-connected linker (Figure 5). The 3D structure of **1** can be simplified as a 12-connected 2-nodal *Au4Ho* topology (Figure 5k) with a point (Schläfli) symbol {4^20^·6^28^·8^18^}{4^3^}_4_ and with a stoichiometry (3-c)_4_(12-c) as analyzed with the TOPOS 4.0 program [86]. The distances between the centers of {Y_4_O_4_} clusters linked by nba^2−^ are 7.94(3) and 18.07(3) Å (Figures S5 and S6 from Supplementary Materials). Interestingly, the entire structure of **1** consisted of innumerable 1D and 2D crossing frameworks in the crystal. From this point of view and as described above, the 3D MOF of **1** also can be classified as a 6-c uninodal *pcu* alpha-Po primitive cubic net with a Schläfli symbol {4^12^·6^3^} (Figure 5f). 

The yttrium compound has 3D frameworks with 1D open channels with an almost square cross-section of 18.07(3) × 18.07(3) Å^2^ running in the [0 0 1] direction that accommodate the coordinated and disordered guest H_2_O and C_2_H_5_OH molecules. This crystal structure presents large square-section channels in which crystallization water and ethanol molecules are localized. These crystallization water and ethanol molecules were not precisely localized by single X-ray diffraction (which explains the bad R indexes and largest diff. peak/hole). However, the overall 3D framework has a 42.8% total potential solvent-accessible area volume of 3880.8 Å^3^ per unit cell volume of 9070.4 Å^3^ as theoretically calculated using PLATON software [87].

**Figure 6:**
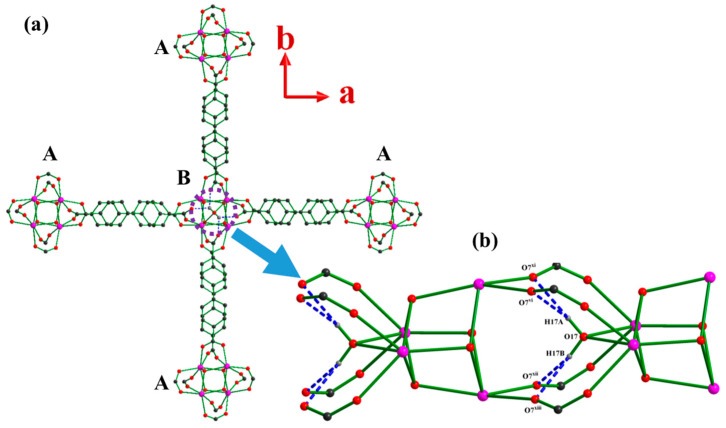
(**a**) The Y1-containing {Y_4_O_4_} core (B) involved in the O-H⋯O hydrogen bond in **1**; (**b**) The enlarged O17-H17A⋯O^vi^, O17-H17B⋯O^xii^ and their symmetry imposed hydrogen bonds interactions in the Y1-containing {Y_4_O_4_} core (B) in **1** (Dash lines represent O-H⋯O hydrogen bonds, symmetry codes see in the Table S3 from Supplementary Materials).

Hydrogen bonding, particularly within the coordination sphere, is another strategy which may lead to enhancement in the chemical stability at the nodes, and therefor the framework materials themselves. There are three types of hydrogen bonding interactions (O-H⋯O, O-H⋯N, and C-H⋯O) calculated by PLATON software in **1** that stabilize its structure (Table S3 from Supplementary Materials). In particular, a 4-centre trifurcated O17-H17A/17B⋯O7^vi^/O7^xi^/O7^xii^/O7^xiii^ hydrogen bond (Figure 6) stabilized the crystal structure of **1**; these were formed between two B clusters: the symmetry-related carboxyl group of L ligand (-O_7_C_15_O_8_-, O7), which is the acceptor, and the *μ*_2_-bridging oxygen of the coordinated water molecule (O7), which acts as the donor (O17⋯O7^vi^/O7^xi^/O7^xii^/O7^xiii^ = 2.88(2) Å, H17A/H17B⋯O7^vi^/O7^xi^/O7^xii^/O7^xiii^ = 2.17 Å, O17-H17A/17B⋯O7^vi^/O7^xi^/O7^xii^/O7^xiii^ = 141.4°). The bond distances of H14⋯O9^xi^/O9^xii^, O13⋯⋯O9^xi^/O9^xii^, H16A⋯N^ii^, and O16⋯N2^ii^ are 2.49 Å, 3.25(2) Å, 2.30 Å, and 3.15(3) Å, which is greater than the sum of the van der Waals radii of H and O, O and O, H and N, O and N, and the bond angles of O13-H13A⋯O2^iv^ and C26-H26⋯O12 are 102° and 101°, which falls slightly below the preferred minimum hydrogen bond angle of 110°. Therefore, they are belonged to a very weak O-H⋯O, O-H⋯N, and C-H⋯O hydrogen bonds according to the document data [88,89,90].

**Figure 7:**
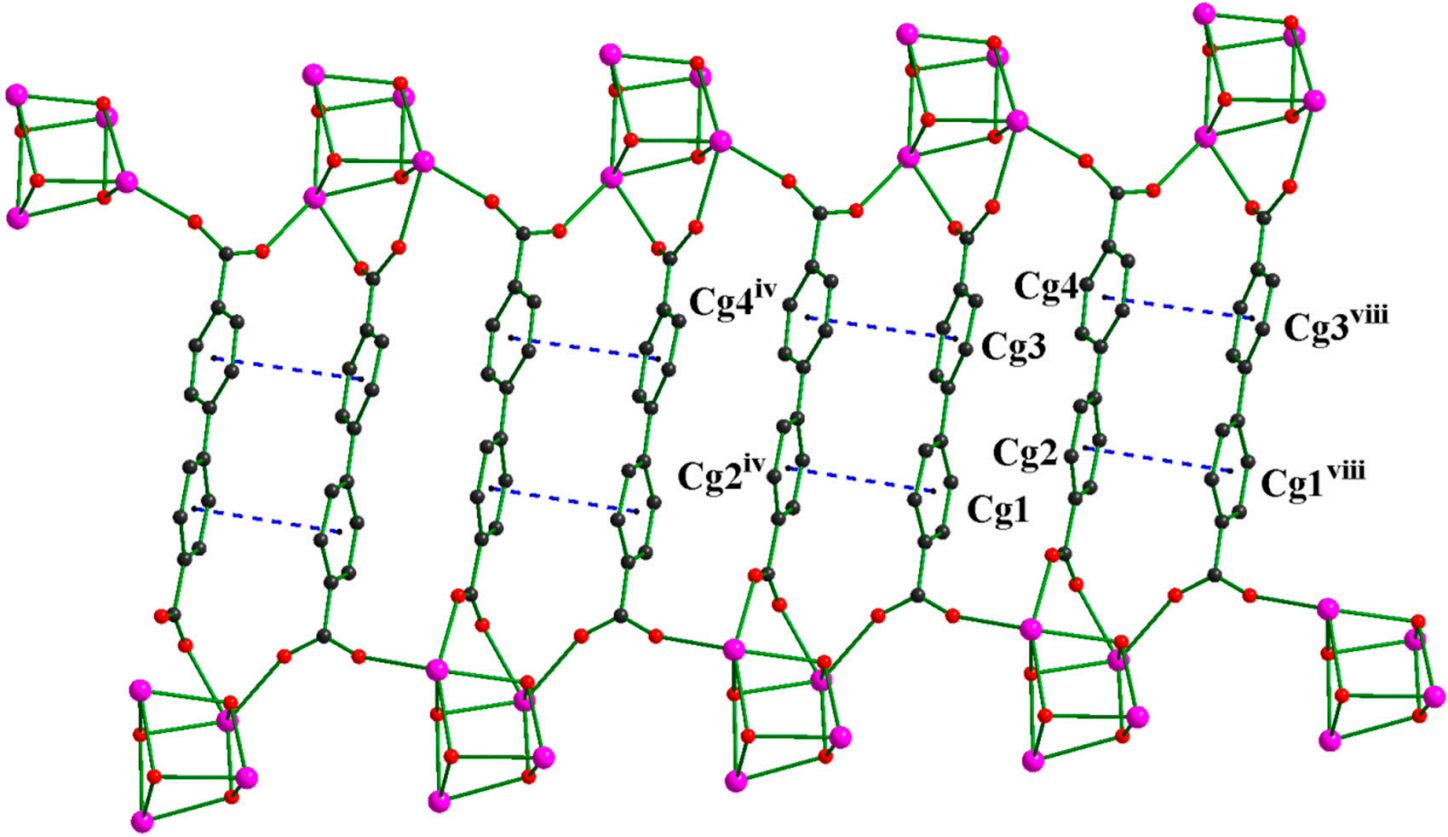
View of the π⋯π interactions in **1** (Blue dash lines represent π⋯π interactions, symmetry codes see in the Table S2 from Supplementary Materials).

In addition, the crystal has a pair of π⋯π stacking interactions (Figure 7): between the four benzene rings (6-membered rings, Cg1: C2/C3/C4/C5/C6/C7, Cg2: C22/C23/C24/C25/C26/C27, Cg3: C8/C9/C10/C11/C12/C13 and Cg4: C16/C17/C18/C19/C20/C21) related to the L ligands. The dihedral angles between the Cg1/Cg2^iv^ or Cg2/Cg1^viii^ and Cg3/Cg4^iv^ or Cg4/Cg3^viii^ (symmetry codes seen in the Table S2 from Supplementary Materials) are 23° or 18.3° and 22.3° or 15.2°, with centroid-to-centroid distances of 3.699(9) Å or 3.586(8) Å and 3.646(9) Å or 3.803(9) Å, and perpendicular distances of 3.586(8) or 3.698(9) Å and 3.804(9) Å or 3.646(9) Å, respectively. The dihedral angles between the two phenyl rings in two bib are 50.59(6)° and 55.29(4)°, respectively, which preventing coplanarity of the rings and weakening π conjugation in **1**. Thus, the three-dimensional network is further stabilized through O-H⋯O, O-H⋯N, C-H⋯O hydrogen bonds and π⋯π interactions.

### 2.2. Luminescent Properties

#### 2.2.1. Fluorescence Properties of **1** and H_2_nba in Aqueous Solution

**Figure 8:**
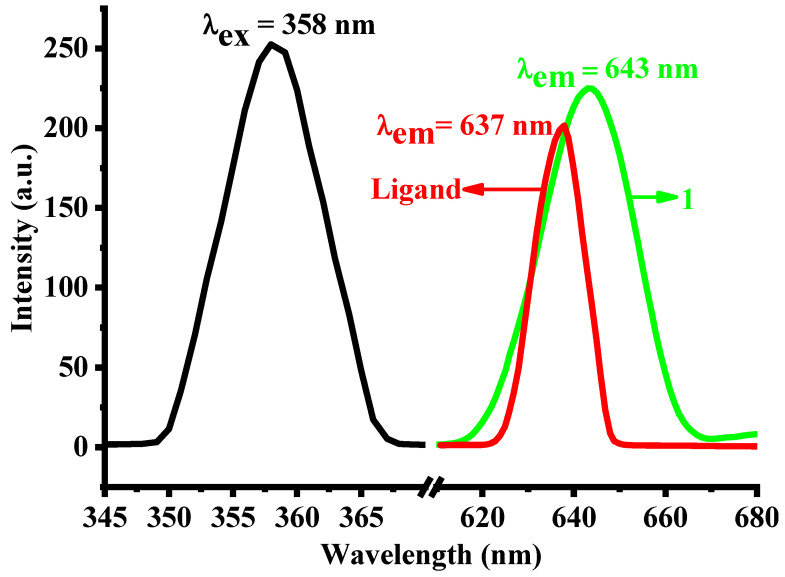
The luminescent properties of **1** and H_2_nba ligand in aqueous solution at room temperature.

Allowing for the presence of tombarthite ion (Y^3+^) in **1** and the large conjugation of the ligand itself, as well as the potential strong interactions between guest molecules and the abundant coordinated polar groups of **1**, we have investigated the luminescent properties of **1** and H_2_nba ligand in aqueous solution at room temperature (Figure 8). Interestingly, MOF **1** and H_2_nba have approximately the same excitation and emission peak shape, except the intensity. The maximum sharp emission peaks are centered at 643 nm (*λ*_ex_ = 358 nm) with the intensity of 225 a.u. for 1, and 637 nm (*λ*_ex_ = 358 nm) with the intensity of 201 a.u. for H_2_nba, which show characteristic red luminescence and may be attributed to π*→n or π*→π transitions. A significant red shift and the emission intensity enlargement appear compared to the emission spectrum of the ligand due to the increased degree of conjugation after the formation of MOF structure, which is often seen in transition metal based MOFs. The maximum emission wavelength showed small shifts after simply dispersing solid **1** samples in various metal salt solvents, while the fluorescence intensity differed more significant under identical concentration. 

#### 2.2.2. Detection of Cations

**Figure 9:**
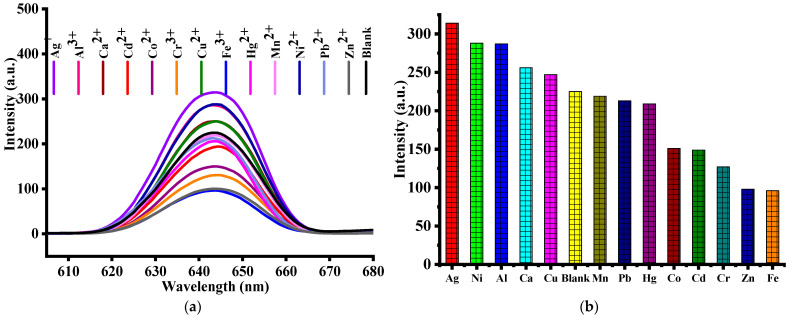
(**a**) Photoluminescence spectra of **1** in water and aqueous solutions containing different metal ions (1 ×10^−3^ M) when excited at 358 nm; (**b**) The photoluminescence intensities of **1** in aqueous solutions with various inorganic cations (1 × 10^−3^ M) when excited at 358 nm.

As shown in the experimental section, a variety of fluorescence spectral measurements have been carried out the fluorescent response of **1** which interacts with 13 different metal cations. **1** (1 mg) was added to aqueous solutions of M(NO_3_)_x_ (1 mL, M = Ag^+^, Ni^2+^, Al^3+^, Ca^2+^, Cu^2+^, Mn^2+^, Pb^2+^, Hg^2+^, Co^2+^, Cd^2+^, Cr^3+^, Zn^2+^, Fe^3+^, x = 1~3) and sonicated for 30 min to form a suspension for fluorescence detection. As illustrated in Figure 9, the maximum fluorescence emission peak of 1 showed different fluorescence intensities for various metal ions and contrasted with blank **1**. Interestingly, we found that the fluorescence of **1** was certainly enhancement by Ag^+^, Ni^2+^, Al^3+^, Ca^2+^, Cu^2+^, moderately quenched by Co^2+^, Cd^2+^, Cr^3+^, Zn^2+^, Fe^3+^, and not quenched at all by the other metal ions (Mn^2+^, Pb^2+^, Hg^2+^). In AgNO_3_ and Fe(NO_3_)_3_ dispersed samples display relatively significant fluorescence intensity shifts compared with referenced blank **1** suspension. The fluorescence intensity was increased 89 a.u. from blank 225 a.u. to 314 a.u. in AgNO_3_, while in Fe(NO_3_)_3_, that value was decreased 129 a.u. from 225 a.u.to 96 a.u.. According to previous reports, the luminescence effect of MOF caused by metal ions mainly results from the destruction of MOF structure, ion exchange, host–guest interaction. Most of the MOFs reported to show sensing behaviors are based on the electron/energy transfer mechanism, which is closely related to the weak interactions (coordination, hydrogen bonds, and π−π stacking) between MOFs and analytes. At present, MOF-based luminescent probes predominately show a fluorescence “turn-off” signal, that is, the emission is quenched, which generally exists in the fluorescence recognition of iron ions owning to the paramagnetic feature and significant visible absorbance of Fe^3+^. On the other hand, analytes also can turn on the fluorescence by limiting the free rotation of the linkers to enhance the conjugation or by breaking the coordination bonds in MOFs to prevent the ligand-to-metal charge transfer or energy transfer. Therefore, development of robust and efficient MOF-derived fluorescence “turn-on” probes for the recognition of metal ions is more meaningful and highly demanded. Al and Ag are typical oxyphilic elements. Therefore, the luminescence enhancement in case of Ag^+^, Ni^2+^ and Al^3+^ ions may be from the interactions between the framework nitro groups with metal ions. Apart from this, it is worth noting that the fluorescence intensity of AgNO_3_ or Fe(NO_3_)_3_ dispersed **1** is only enhanced or reduced by about 39.56% or 57.33% compared with that of the referenced **1** dispersed sample. In addition, in the fluorescent metal cations recognition experiments for **1**, it is further found that the fluorescence of **1** in AgNO_3_ or Fe(NO_3_)_3_ aqueous solution is insensitive and the effect is not ideal, so we take the anions fluorescence recognition of 1 as an example to discuss in detail in this article.

#### 2.2.3. Detection of F^−^ Anion

**Figure 10:**
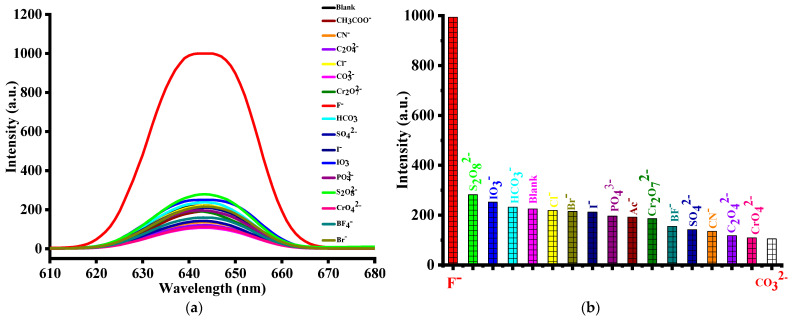

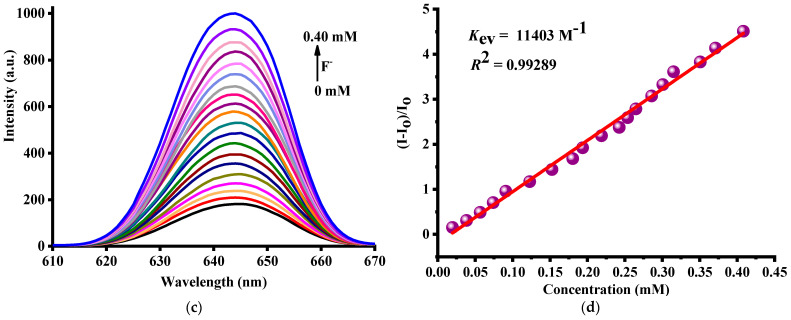
(**a**) Photoluminescence spectra of **1** in water and aqueous solutions containing different anions (1 × 10^−3^ M) when excited at 358 nm; (**b**) The photoluminescence intensities of **1** in aqueous solutions with various anions (1 × 10^−3^ M) when excited at 358 nm; (**c**) The emission spectra for **1** in aqueous solution of different F^−^ concentrations; (**d**) The linear relationships for **1** in aqueous solution of different F^−^ concentrations.

Besides metal cations, in order to improve the use efficiency of **1** as a multifunctional fluorescent probe, the fluorescence recognition anion experiments were performed to screen 16 sodium salt anions in aqueous solution (Figure 10a,b), with the concentration of the anions being 1 mM. Outstandingly, only F^−^ afforded a quite obvious luminescent turn-on effect, with a turn-on percentage of 344.4%, but the enhancement or quenching percentage of other anions was much lower (less than 25% for S_2_O_8_^2−^, 53% for CO_3_^2−^) under the same conditions. It was also found that only slight enlargement or reduction changes occurred when detecting IO_3_, or PO_4_^3−^, Ac^−^, Cr_2_O_7_^2−^, BF_4_^−^, SO_4_^2−^, CN^−^, C_2_O_4_^2−^, CrO_4_^2−^, while HCO_3_^−^, Cl^−^, Br^−^ and I^−^ had almost no fluorescence intensity changes.

Quantitative experiments were then carried out with 1 suspension selected as reference and NaF solution was increasingly added to monitor the emissive response. On account of the distinct response of **1** toward F^−^, the turn on effect of F^−^ was subsequently explored with a function of NaF in the concentration range of 0~0.40 mM. As shown in Figure 10c, when the F^−^ concentration was gradually raised from 0 to 0.40 mM, the fluorescence intensity from 225 a.u. promoted little by little to about 1000 a.u., and the fluorescence intensity significantly increased by nearly 4.5 times when the concentration of the F^−^ ions reached 0.40 mM, exhibiting very strong fluorescence response.

In order to quantitatively analyze the relationship between the luminescence intensity of 1 and the content of NaF, we defined a luminescence enhancement coefficient K_ec_ according to the equation *I*/*I*_0_=1+*K*_ec_[M], where [M] is the molar concentration of the NaF, I represents the luminescence intensity of **1** suspension after adding NaF up to specified concentration, and I_0_ is the initial luminescence intensity of 1 suspension. As shown in Figure 10d, it is clear that in a wide concentration range from 0.02 to 0.40 mM, the relative intensity has a good linear relationship with the F^−^ concentration. The calculated stern-volmer constant *K*_ec_ value is 11403 M^−1^, which indicates that **1** has a high sensitivity for fluor ion detection. Moreover, the detection limit (LOD) of F^−^ is 0.26 ppm (14.20 µM) based on formula 3*σ*/slope (*R*^2^ = 0.99289, *σ* = 0.0539), which is far less than the World Health Organization (WHO) (1.50 ppm, 81.92 µM) or the U.S. Environmental Protection Agency (EPA) (3.85 ppm, 210 µM) guideline for the limit of the maximum F^−^ concentration in drinking water. Notably, such a small LOD value is much lower than most MOF-based F^−^ fluorescent probes. Furthermore, the fluorescence sensing capabilities of **1** and **1**@F^−^ were also evaluated at different pH values (0-14). As shown in Figure S7 (from Supplementary Materials), The fluorescence emission of **1** and **1**@F^−^ have slight or no discernible intensity change observed in the range of pH = 4–13, while a moderate intensity decrease for **1** and increase for **1**@F^−^ was found in the case of pH = 2, 3 and 14. It is noteworthy that the most acidic solution led to the biggest fluorescence intensity changes for **1** or **1**@F^−^ (pH = 0 and 1). These results imply that **1** could serve as a fluorescent probe for F^−^ under complex physiological pH conditions and could be highly selective for sensing F^−^ in actual environments. The above results also implying **1** exhibits selective turn-on effects toward F^−^ and can be used as a quantitative recognition F^−^ probe in aqueous solution.

In our opinion, the continuous intramolecular hydrogen bonds distribute in **1** should be critical for such an excellent detection performance. If the molecular structure contains hydrogen bond donor (-OH or –NH_2_) and hydrogen bond receptor (-NO_2_ and -COO), then such molecules can undergo intramolecular proton transfer [35,91,92,93]. On the another hand, as the smallest anion with a strong electronegativity, fluoride ions readily form hydrogen bonds with hydrogen atoms in the hydrogen bond donor, and thus influences the process of the excited state intramolecular proton transfer (ESIPT) fluorophore. So, it is anticipated that the introduction of ESIPT into MOFs should be promising for high-efficient fluorescence sensing of fluoride ions. Such a high selectivity ability of fluorescence sensing process for F^−^ may be due to the hydrogen bonds between F^−^ and coordinated H_2_O or μ_3_-bridged hydroxyl groups OH^−^ is strongest and the influence of other anions is negligible.

#### 2.2.4. Stability and Cyclic Use Test

**Figure 11:**
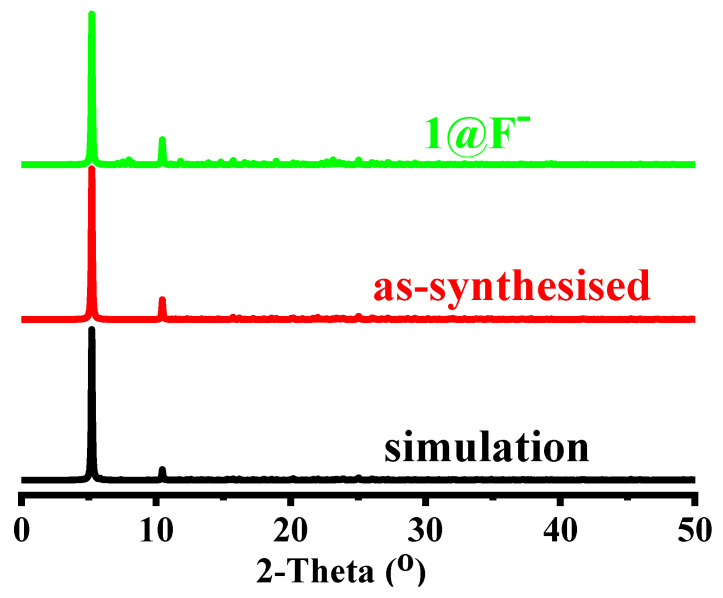
PXRD profile of the as-synthesized **1** and the simulated result as reference as well as luminescence sensing analytes.

The powder X-ray diffraction (PXRD) pattern of **1** is shown in Figure 11. The experimental peaks are consistent with the theoretical one obtained by single-crystal X-ray data. To confirm the phase purity and stability, **1** was treated with NaF aqueous solution at room temperature. Then, the MOF powders were collected and dried in air after **1** was soaked in these solutions for 12 h. The powder X-ray diffraction (PXRD) measurements were carried out to study the structures of the original and anion incorporated MOF samples of **1**@F^−^. The PXRD pattern of the **1**@F^−^ is similar to that of **1**, respectively, ruling out the collapse of the MOF frameworks. The slight variation of diffraction intensity may be related to the different crystal orientation or solvent effects. Although the experimental patterns have a few unindexed diffraction lines and some are slightly broadened in comparison to those simulated from single-crystal model, it can still to be considered that the bulk synthesized materials and as-grown crystal are homogeneous, implying its excellent stability.

In order to explore the thermal stability of **1**, TG studies have been performed in a nitrogen atmosphere at a heating rate of 10 °C min^−1^ between 50 and 950 °C (Figure S8 from Supplementary Materials). Up to 165 °C, almost no weight loss was observed on the TG curve. For **1**, a weight loss of 3.82% was occurred at 160–220 °C, which corresponds to the loss of coordination water molecules and remaining crystallized or disordered guest molecules in the pores (calcd. 4.31%). According to TG data, heating above 320 °C leads to several different processes, which lead to the decomposition of the framework of compounds **1**. This result suggested the coordination framework was thermally stable below 320 °C The subsequent large weight loss could be ascribed to the loss of coordination hydroxide radical and decomposition of organic ligands, and destruction of coordination bonds. The final white residues were about 28.25% (calcd. 28.40%) of the initial total weight for **1**, which may be Y2O_3_.

**Figure 12:**
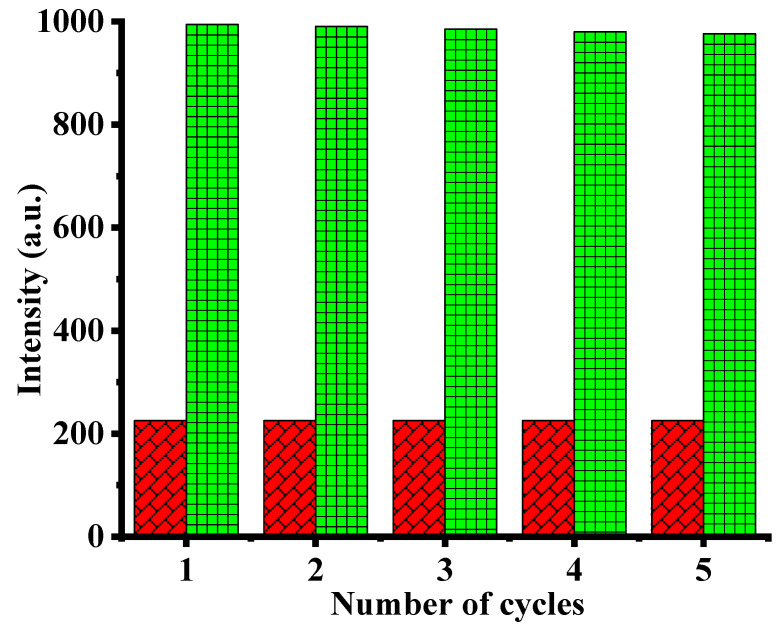
Photoluminescence intensity of **1** in five recyclable experiments for the F^−^ in aqueous solution (The red bar shown the initial fluorescence intensity, the green bar shown the intensity upon addition a distilled water of 1mM F^−^ anion).

Repeatability is an important factor for assessing the applicability of a luminescent coordination polymers. Recyclability experiments were conducted to find out the regeneration of **1** to detect F ^−^ anion. The used sample of **1** was washed multiple times with alcohol through soaking under stirring at room temperature. After filtration and drying, the regenerated sample **1** was employed for the detection of F ^−^ anion with the same way of **1**, respectively. It is obvious that the quenching ability of **1** for F ^−^ anion, respectively, are mostly unchanged up to five cycle (Figure 12). All these results indicate **1** has excellent water stability and reusable value.

#### 2.2.5. IR Spectrum Analysis

IR spectra of carboxylate complexes can provide information to distinguish the coordination modes of the carboxyl groups. As shown in Figure S9 (from Supplementary Materials), **1** and **1**@F^−^ exhibit broad absorption bands in the range 3700–2800 cm^−1^ assigned to the O-H characteristic stretching vibration (ν(OH)) from the coordinated hydroxyl groups (μ_3_-OH) and the coordinated water molecules. Comparison with **1** and **1**@F^−^, the infrared spectrums of H_2_nba ligand is lack of *ν*(OH) absorption bands at about 3000 cm^−1^. On the other hand, comparison with the H_2_nba (*ν*(COOH) = 1680 cm^−1^) ligand, the infrared spectrums of **1** and **1**@F^−^ are obvious absence of *ν*(COOH) absorption peaks about 1700 cm^−1^, which indicates the complete deprotonation of H_2_nba after the two carboxylate O atoms coordinated to Y^3+^ ions in **1** and **1**@F^−^. In addition, the IR spectrum of H_2_nba, **1**, and **1**@F^−^ show a series of similar characteristic strong bands range from 1610 to 600 cm^−1^. The absorption bands at 1609 cm^−1^ and 657 cm^−1^ for H_2_nba, 1600 cm^−1^ and 652 cm^−1^ for **1**, and 1609 cm^−1^ and 647 cm^−1^ for **1**@F^−^, are due to υ(C=C) of benzene of ligands. The peaks at ca. 768 m^−1^ for H_2_nba, 764 m^−1^ for **1**, and 773 cm^−1^ for **1**@F^−,^ are due to the out-of-plane C–H bending vibration. Bands assigned to *ν*_as_(COO^–^) and *ν*_s_(COO^–^), which are observed for the free H_2_dba ligand at ca. 1538 cm^−1^ and 1285 cm^−1^, respectively, are shifted to 1535 cm^−1^ for **1**, 1529 cm^−1^ for **1**@F^−^ and 1366 for **1**, 1361 cm^−1^ for **1**@F^−^, indicating that deprotonation of the carboxylate group occurred upon coordination. The difference between the asymmetric and symmetric stretches, Δ(*ν*_as_(COO^–^) − *ν*_s_(COO^–^)) is more smaller than 200 cm^−1^ (253 cm^−1^ for H_2_nba, 172 cm^−1^ for **1**, and 177 cm^−1^ for **1**@F^−^, respectively), indicating the bridging mode of the carboxylate groups found in **1** and **1**@F^−^. The low intensity bands in the region of 600–400 cm^−1^ are attributed to Y–O vibration for **1** and **1**@F^−^. All these spectroscopic features of H_2_nba, **1**, and **1**@F^−^ are consistent with the crystal structure determinations. Interestingly, the significant different FT-IR spectra absorption bands in the range 3700–2800 cm^−1^ for **1** and **1**@F^−^ indicate that the intensity of the hydroxyl peak (3244 cm^−1^) weakens after the addition of F^−^, the latter splitted into 2 small peaks (3381 and 3123 cm^−1^) and becomes very weak, which may be accounted for the interactions of hydroxyl groups with F^−^ ions and further support the formation of hydrogen bonds between fluoride ion and hydroxyl group in the fluorescence turn on recognition process.

#### 2.2.6. The Scalability of the Synthesis for **1**

Y-based MOFs are usually synthesized using DMF as a solvent and 2-fba acid as a modulator. In this work we follow a diverse synthetic route resulting however at a tetranuclear cluster [Y_4_(OH)_4_]^8+^ instead of an 1D chain, which is usual for water based syntheses. This route consists of using lanthanide nitrate as a starting material and in hydrolyzing it by the addition of sodium hydroxide. This synthetic pathway is based on a subtle balance between the various experimental parameters (pH, concentrations, temperature, etc.). The main difficulty encountered during the syntheses of these complexes is to avoid the formation of very stable polymeric species such as Ln(OH)_2_NO_3_, LnONO_3_, and Ln(OH)_3_. In fact, in the synthesis experiments of MOF-**1**, we have tried various experimental parameters (DMF as a solvent and 2-fba acid as a modulator, metal salts, pH, concentrations, temperature, etc.) and some other factors for corresponding synthesis experiments. However, only for Y elements, single crystals appeared according to the reported assembly processes. In addition, a series of MOFs single crystals formed by transition metal salts (still under further study) with above experimental parameters enables us to further explore the relationship between MOFs structures and solvents or modulators or neutral auxiliary ligands.

## 3. Materials and Methods

### 3.1. Chemicals and Reagents

All chemicals and solvents including the ligands were purchased from Jinan Henghua Sci. & Tec. Co. Ltd. without further purification.

### 3.2. Apparatus

Powder X-ray diffraction (PXRD) data were collected on a Bruker D8 ADVANCE X-ray diffractometer (Karlsruhe, Germany) with Cu-Kα radiation (λ = 1.5418 Å) at 40 kV, 40 mA with a scanning rate of 6°/min and a step size of 0.02°. The simulation of the PXRD spectra was carried out by the single-crystal data and diffraction-crystal module of the Mercury 2.0 (Hg) program available free of charge via the Internet at http://www.iucr.org (accessed on 25 January 2023). The purity and homogeneity of the bulk products were determined by comparing the simulated and experimental X-ray powder diffraction patterns. The elemental analyses (C, H, N) were performed on a Perkin-Elmer 240C apparatus (Norwalk, CT, USA). FT-IR spectra were recorded in the range of 4000–450 cm^−1^ on a PerkinElmer Frontier spectrometer (Norwalk, CT, USA). Thermogravimetric analyses (TG) were performed under nitrogen with a heating rate of 10 °C min^−1^ using a PerkinElmer Thermogravimetric Analyzer TGA4000 (Norwalk, CT, USA). Photoluminescence spectra were measured on a Varian Cary Eclipse fluorescence spectrophotometer (Shanghai, China) with a xenon arc lamp as the light source. In the measurements of emission and excitation spectra, the slit width is 10 nm. Point symbol and topological analyses were conducted by using the TOPOS program package.

Single-crystal data collections were performed on a XtaLAB Mini (ROW) diffractometer (Tokyo, Japan) with graphite-monochromatized Mo*K*α radiation (*λ* = 0.71073 Å). Each crystal was kept at T = 298.15 K during data collection. Using Olex2 [94], the structure was solved with the ShelXT structure solution program using Intrinsic Phasing [95] and refined with the ShelXL refinement package using Least Squares minimization [96]. All non-hydrogen atoms were refined with anisotropic displacement parameters. Carbon-bound H-atoms were placed in calculated positions (*d*_C-H_ = 0.93 Å for -CH) and were included in the refinement in the riding model approximation, with *U*_iso_(H) set to 1.2*U*_eq_(C) for -CH. The H atoms of coordinated H_2_O and *μ*_3_-OH^−^ hydroxyl groups in **1** were refined as rotating groups, with *d*_O–H_ = 0.85 Å and *U*_iso_(H) = 1.5*U*_eq_(O). Two coordinated molecules in the B cluster of **1** are disordered. They were both refined as distributed on two limit positions fixing to the site occupancy of 0.25 for O17 and 0.5 for O16. There is a large solvent accessible pore volume in the structure of 1, which is occupied by highly disordered solvent molecules. No satisfactory disorder model for these solvent molecules could be achieved, and therefore the SQUEEZE program implemented in PLATON was used to remove these electron densities of these disordered species. The structures were examined using the Addsym subroutine of PLATON to ensure that no additional symmetry could be applied to the models. Pertinent crystallographic data collection and refinement parameters are collated in Table S1 (from Supplementary Materials). Selected bond lengths and angles are given in Table S2 (from Supplementary Materials). Hydrogen-bond geometry are organized in Table S3 (from Supplementary Materials).

CCDC 2237853 (**1**) contains the supplementary crystallographic data for this paper. These data can be obtained free of charge from The Cambridge Crystallographic Data Centre via http://www.ccdc.cam.ac.uk/data request/cif (accessed on 25 January 2023).

### 3.3. Synthesis of {[Y(μ_3_-OH)]_4_[Y(μ_3_-OH)(μ_2_-H_2_O)_0.25_(H_2_O)_0.5_]_4_[μ_4_-nba]_8_}_n_ (**1**) 

A mixture of Y(NO_3_)_3_·8H_2_O (0.1 mmol), H_2_nba (0.1 mmol), NaOH (0.1 mmol), and H_2_O-C_2_H_5_OH (5 mL/5 mL) was added to a 25 mL Teflon-lined stainless steel reactor and heated at 120 °C for 3 days, and then slowly cooled to room temperature. Light yellow block single crystals suitable for X-ray data collection were obtained by filtration. Yield: 35% (based on yttrium nitrate octahydrate) —Anal. for C_112_H_70_N_8_O_59_Y_8_ (3183.04): calcd. C 42.22, H 2.20, N 3.52; found C 42.25, H 2.18, N 3.50. Infrared (KBr pellet, cm^−1^): 3244 (w), 1600 (m), 1535 (m), 1439 (m), 1366 (s), 1313 (w), 1247 (w), 1140 (w), 1017 (w), 842 (m), 768 (m), 734 (w), 652 (m).

### 3.4. Photoluminescent Sensing Experiments

The luminescence properties of **1** have been investigated in the aqueous solution of various analytes at room temperature [97,98]. When measuring the emission spectra, the emission and excitation slit width were set as 5 nm and 2.5 nm (similarly hereinafter), respectively. For sensing of cations, 1.0 mg of a grounded powder samples of **1** was immersed in 1.0 mL 13 cations aqueous solution of M(NO_3_)_x_ (M^x+^ = Ag^+^, Ni^2+^, Al^3+^, Ca^2+^, Cu^2+^, Mn^2+^, Pb^2+^, Hg^2+^, Co^2+^, Cd^2+^, Cr^3+^, Zn^2+^, and Fe^3+^, 0.0010 mol•L^−1^). Then the solid-liquid mixture was ultrasonicated for 30 min to form steady turbid suspension of **1**@M^x+^ for the fluorescence measurements. The fluorescent intensities of these **1**@M^x+^ suspensions were immediately recorded at room temperature and compared. For sensing of 16 anions, the same procedure was used to the **1**@A^y−^ fluorescence measurements (Na_y_A, A^y−^ = F^−^, S_2_O_8_^2−^, IO_3_, HCO_3_^−^, Cl^−^, Br^−^, I^−^, PO_4_^3−^, Ac^−^, Cr_2_O_7_^2−^, BF_4_^−^, SO_4_^2−^, CN^−^, C_2_O_4_^2−^, CrO_4_^2−^, and CO_3_^2−^, 0.0010 mol•L^−1^). The fluorescence intensity of the suspension **1** in water was measured as the blank sample.

### 3.5. Fluorescence Titration Experiments for F^−^

The 1.0 mg grounded powder sample of **1** was dispersed in 1.0 mL H_2_O solution of target analyte NaF with different concentrations (0.02~0.40 mM). Then the solid-liquid mixture was ultrasonicated for 30 min to form steady turbid suspension of **1**@F^−^ for the fluorescence measurements. The fluorescent intensities of these **1**@F^−^ suspensions were immediately recorded at room temperature and compared. 

### 3.6. Recyclable Luminescence Experiments

The powder of complex **1** was centrifuged from the suspension and rinsed with distilled water for three times. Then, the recovered sample was dried and added to the target analytes again to measure their emission spectra. The same procedure was repeated five times.

## 4. Conclusions

In summary, a new Y(III)-based metal-organic framework derived from the H_2_nba ligand has been hydrothermally synthesized and characterized by IR spectroscopy, elemental analysis and single-crystal X-ray diffraction. The topological structure analysis revealed that **1** is a 3D MOF with a 12-connected *Au4Ho* topology with a Schläfli symbol {4^20^·6^28^·8^18^}{4^3^}_4_ or a 6-connected *pcu* topology with a Schläfli symbol {4^12^·6^3^} net. The calculated detection limit of **1** for F^−^ ion was found to be 14.2 μM, which is much better than most of the reported results and much lower than the permissible amount in water as recommended by the WHO or EPA. The fluorescence detection process of **1** for F^−^ was proposed to base on ESIPT mechanism. These existing results may provide a new platform for designing luminous Ln-MOFs with application for detecting F^−^ in drinking water in the future.

## Data Availability

Not applicable.

## Supplementary Materials

The following are available online at https://www.mdpi.com/article/10.3390/molecules28041893/s1, Figure S1. The asymmetry unit in **1**; Figure S2. The coordination configurations for Y1, Y2 and Y3 ions in **1**; Figure S3. (a–c) The {Y1}_4_ or {O}_4_ constitutes a tetrahedron in **1**; (d–g) The {Y2-Y3}_4_ or {O}_4_ constitutes a tetrahedron in **1**; Figure S4. (a–d) View of the 3D simplified schematic diagram of **1** and the 3D framework with 1D open channels running in the [0 0 1] direction; Figure S5. (a) View one of the nba^2−^ ligand connected with two B and one A clusters in **1**; (b) The simplied ligand 3-connected node; Figure S6. (a) View one of the nba^2−^ ligand connected with two A and one B clusters in **1**; (b) The simplied ligand 3-connected node; Figure S7. Fluorescence intensity of **1** in different pH aqueous solution with the unintroduction ions (red) and introduction of F^−^ (magenta); Figure S8. TG curves of compound **1**; Figure S9. The infrared spectra of H2nba ligand, **1** before and after F^−^ sensing. Table S1. Crystal structure data for **1**^a,b,c^; Table S2. Selected bond lengths/Å and bond angles/º for compound **1**; Table S3. Selected bond lengths (Å) and angles (deg) for **1** with estimated standard deviations in parentheses. File S1: checkCIF/PLATON report.

## Sample Availability

Samples of the compounds are not available from the authors.
