# Supplementary material for: A 12-Connected [Y4((μ3-OH)4]8+ Cluster-Based Luminescent Metal-Organic Framework for Selective Turn-on Detection of F− in H2O
Source: Molecules. 2023 Feb 16;28(4):1893. doi: 10.3390/molecules28041893 (PMC9960892; doi:10.3390/molecules28041893)

checkCIF/PLATON report

Structure factors have been supplied for datablock(s) 190610c_sq

THIS REPORT IS FOR GUIDANCE ONLY. IF USED AS PART OF A REVIEW PROCEDURE FOR PUBLICATION, IT SHOULD NOT REPLACE THE EXPERTISE OF AN EXPERIENCED CRYSTALLOGRAPHIC REFEREE.

No syntax errors found.   CIF dictionary   Interpreting this report

Datablock: 190610c_sq

```
Bond precision:  C-C = 0.0253 A          Wavelength=0.71073

Cell:            a=33.7993(10)     b=33.7993(10)     c=7.9398(5)
                 alpha=90          beta=90           gamma=90
Temperature:     298 K
```

	Calculated	Reported
Volume	9070.4(8)	9070.4(8)
Space group	P -4 21 m	P -4 21 m
Hall group	P -4 2ab	P -4 2ab
Moiety formula	C112 H70 N8 O59 Y8 [+ solvent]	C112 H70 N8 O59 Y8
Sum formula	C112 H70 N8 O59 Y8 [+ solvent]	C112 H70 N8 O59 Y8
Mr	3183.04	3183.04
Dx,g cm-3	1.166	1.165
Z	2	2
Mu (mm-1)	2.598	2.598
F000	3164.0	3164.0
F000’	3121.15	
h,k,lmax	40,40,9	40,40,9
Nref	8308[4625]	8216
Tmin,Tmax	0.541,0.677	0.262,1.000
Tmin’	0.517	

```
Correction method= # Reported T Limits: Tmin=0.262 Tmax=1.000
AbsCorr = MULTI-SCAN

Data completeness= 1.78/0.99      Theta(max)= 25.100

R(reflections)= 0.1209( 6343)     wR2(reflections)= 0.3291( 8216)

S = 1.053                   Npar= 178
```

The following ALERTS were generated. Each ALERT has the format
test-name_ALERT_alert-type_alert-level.
Click on the hyperlinks for more details of the test.

Alert level B

```
DIFMN02_ALERT_2_B  The minimum difference density is < -0.1*ZMAX*1.00
            _refine_diff_density_min given =     -4.683
            Test value =     -3.900
PLAT098_ALERT_2_B Large Reported Min.  (Negative) Residual Density      -4.68 eA-3
PLAT341_ALERT_3_B Low Bond Precision on  C-C Bonds ...............    0.02533 Ang.
PLAT420_ALERT_2_B D-H Without Acceptor       O15      --H15      .     Please Check
PLAT420_ALERT_2_B D-H Without Acceptor       O16      --H16B     .     Please Check
```

Alert level C

```
DIFMN03_ALERT_1_C  The minimum difference density is < -0.1*ZMAX*0.75
            The relevant atom site should be identified.
DIFMX02_ALERT_1_C  The maximum difference density is > 0.1*ZMAX*0.75
            The relevant atom site should be identified.
STRVA01_ALERT_4_C           Flack test results are ambiguous.
           From the CIF: _refine_ls_abs_structure_Flack    0.490
           From the CIF: _refine_ls_abs_structure_Flack_su    0.030
PLAT082_ALERT_2_C High R1 Value ..................................       0.12 Report
PLAT084_ALERT_3_C High wR2 Value (i.e. > 0.25) ....................       0.33 Report
PLAT097_ALERT_2_C Large Reported Max.  (Positive) Residual Density       3.59 eA-3
PLAT241_ALERT_2_C High   'MainMol' Ueq as Compared to Neighbors of         O1 Check
PLAT241_ALERT_2_C High   'MainMol' Ueq as Compared to Neighbors of         O5 Check
PLAT241_ALERT_2_C High   'MainMol' Ueq as Compared to Neighbors of         O6 Check
PLAT241_ALERT_2_C High   'MainMol' Ueq as Compared to Neighbors of         O7 Check
PLAT241_ALERT_2_C High   'MainMol' Ueq as Compared to Neighbors of        O15 Check
PLAT242_ALERT_2_C Low    'MainMol' Ueq as Compared to Neighbors of         Y1 Check
PLAT242_ALERT_2_C Low    'MainMol' Ueq as Compared to Neighbors of         Y2 Check
PLAT242_ALERT_2_C Low    'MainMol' Ueq as Compared to Neighbors of         Y3 Check
PLAT250_ALERT_2_C Large U3/U1 Ratio for Average U(i,j) Tensor ....        2.6 Note
PLAT369_ALERT_2_C Long   C(sp2)-C(sp2) Bond  C25      - C28      .       1.55 Ang.
PLAT910_ALERT_3_C Missing # of FCF Reflection(s) Below Theta(Min).          6 Note
```

Alert level G

```
PLAT002_ALERT_2_G Number of Distance or Angle Restraints on AtSite         40 Note
PLAT003_ALERT_2_G Number of Uiso or Uij Restrained non-H Atoms ...         50 Report
PLAT007_ALERT_5_G Number of Unrefined Donor-H Atoms ..............          7 Report
PLAT072_ALERT_2_G SHELXL First  Parameter in WGHT  Unusually Large       0.19 Report
PLAT083_ALERT_2_G SHELXL Second Parameter in WGHT  Unusually Large      83.22 Why ?
PLAT171_ALERT_4_G The CIF-Embedded .res File Contains EADP Records          3 Report
PLAT172_ALERT_4_G The CIF-Embedded .res File Contains DFIX Records         42 Report
PLAT174_ALERT_4_G The CIF-Embedded .res File Contains FLAT Records          4 Report
PLAT177_ALERT_4_G The CIF-Embedded .res File Contains DELU Records          1 Report
PLAT300_ALERT_4_G Atom Site Occupancy of O16       Constrained at         0.5 Check
PLAT300_ALERT_4_G Atom Site Occupancy of H16A      Constrained at         0.5 Check
PLAT300_ALERT_4_G Atom Site Occupancy of H16B      Constrained at         0.5 Check
PLAT300_ALERT_4_G Atom Site Occupancy of H17A      Constrained at         0.5 Check
PLAT300_ALERT_4_G Atom Site Occupancy of H17B      Constrained at         0.5 Check
PLAT301_ALERT_3_G Main Residue  Disorder ..............(Resd  1  )         1% Note
PLAT606_ALERT_4_G Solvent Accessible VOID(S) in Structure ........          ! Info
PLAT789_ALERT_4_G Atoms with Negative _atom_site_disorder_group  #          3 Check
PLAT794_ALERT_5_G Tentative Bond Valency for Y1        (III)      .       2.93 Info
PLAT804_ALERT_5_G Number of ARU-Code Packing Problem(s) in PLATON           9 Info
PLAT860_ALERT_3_G Number of Least-Squares Restraints .............        194 Note
PLAT869_ALERT_4_G ALERTS Related to the Use of SQUEEZE Suppressed           ! Info
```

```
PLAT870_ALERT_4_G ALERTS Related to Twinning Effects Suppressed ..          ! Info
PLAT883_ALERT_1_G No Info/Value for _atom_sites_solution_primary .     Please Do !
PLAT909_ALERT_3_G Percentage of I>2sig(I) Data at Theta(Max) Still       50% Note
PLAT913_ALERT_3_G Missing # of Very Strong Reflections in FCF ....          1 Note
```

```
   0 ALERT level A = Most likely a serious problem - resolve or explain
   5 ALERT level B = A potentially serious problem, consider carefully
  17 ALERT level C = Check. Ensure it is not caused by an omission or oversight
  25 ALERT level G = General information/check it is not something unexpected

   3 ALERT type 1 CIF construction/syntax error, inconsistent or missing data
  20 ALERT type 2 Indicator that the structure model may be wrong or deficient
   7 ALERT type 3 Indicator that the structure quality may be low
  14 ALERT type 4 Improvement, methodology, query or suggestion
   3 ALERT type 5 Informative message, check
```

It is advisable to attempt to resolve as many as possible of the alerts in all categories. Often the minor alerts point to easily fixed oversights, errors and omissions in your CIF or refinement strategy, so attention to these fine details can be worthwhile. In order to resolve some of the more serious problems it may be necessary to carry out additional measurements or structure refinements. However, the purpose of your study may justify the reported deviations and the more serious of these should normally be commented upon in the discussion or experimental section of a paper or in the "special_details" fields of the CIF. checkCIF was carefully designed to identify outliers and unusual parameters, but every test has its limitations and alerts that are not important in a particular case may appear. Conversely, the absence of alerts does not guarantee there are no aspects of the results needing attention. It is up to the individual to critically assess their own results and, if necessary, seek expert advice.

Publication of your CIF in IUCr journals

A basic structural check has been run on your CIF. These basic checks will be run on all CIFs submitted for publication in IUCr journals (*Acta Crystallographica*, *Journal of Applied Crystallography*, *Journal of Synchrotron Radiation*); however, if you intend to submit to *Acta Crystallographica Section C* or *E* or *IUCrData*, you should make sure that full publication checks are run on the final version of your CIF prior to submission.

Publication of your CIF in other journals

Please refer to the *Notes for Authors* of the relevant journal for any special instructions relating to CIF submission.

PLATON version of 05/12/2020; check.def file version of 05/12/2020

Datablock 190610c_sq - ellipsoid plot

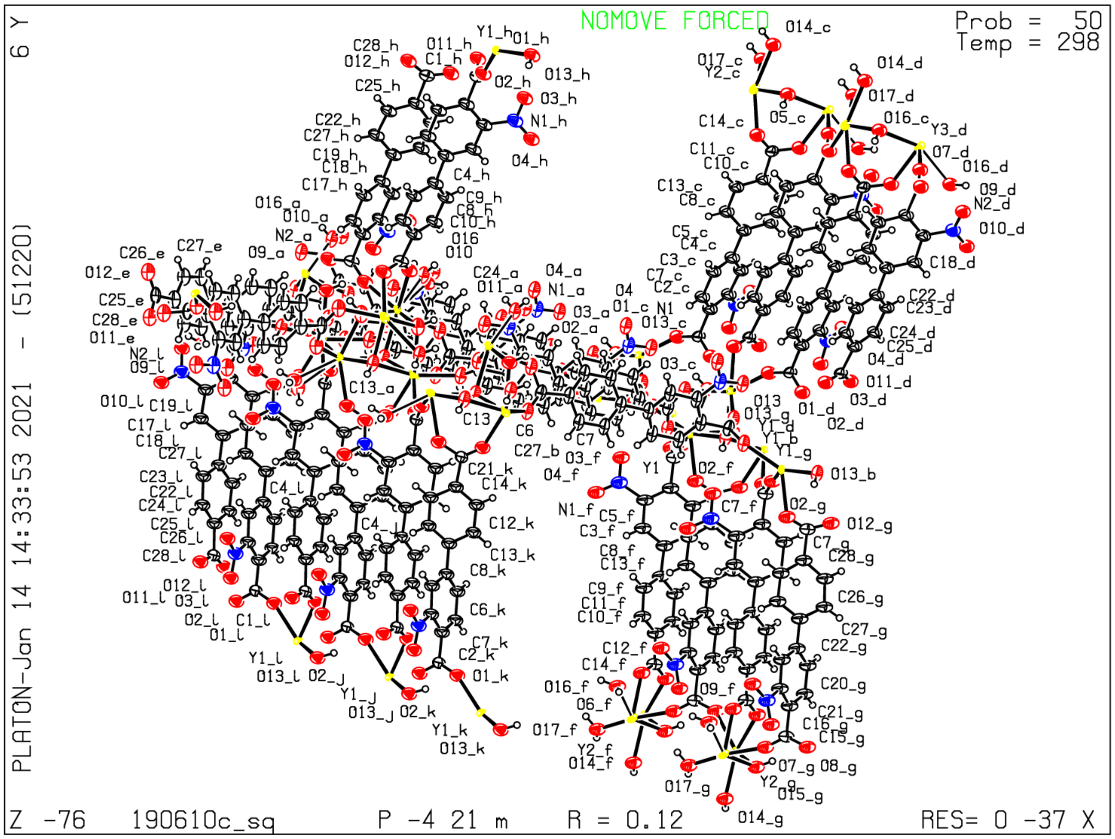

Supplement: Supplementary file 1 [file molecules-28-01893-s001.zip › 1-check CIF.pdf]
