# Supplementary material for: A 12-Connected [Y4((μ3-OH)4]8+ Cluster-Based Luminescent Metal-Organic Framework for Selective Turn-on Detection of F− in H2O
Source: Molecules. 2023 Feb 16;28(4):1893. doi: 10.3390/molecules28041893 (PMC9960892; doi:10.3390/molecules28041893)
Supplement: Supplementary file 1 [file molecules-28-01893-s001.zip › molecules-2209461-supplementary.pdf]

Supplementary Material

A 12-Connected $[Y_4(\mu_3\text{-}OH)_4]^{8+}$ Cluster-Based Luminescent Metal-organic Framework for Selective Turn-on Detection of F^- in H_2O

Juan Li[1, #], Ai-Rong Wang [1, *, #], Shi-Ming Qiu [2, *], Xiao-Li Wang [1], and Jia-Ming Li [1,*]

[1] Guangxi Key Laboratory of Green Chemical Materials and Safety Technology, College of Petroleum and Chemical Engineering, Beibu Gulf University, Qinzhou 535011, China
[2] College of Chemistry and Biological Engineering, Guangxi Minzu Normal University，Chongzuo 532200, China
* Correspondence: wangairong@bbgu.edu.cn (A.-R.W.); qiushiming08@mails.ucas.ac.cn (S.-M.Q.); jmli@bbgu.edu.cn (J.-M.L.)
[#] Juan Li and Ai-Rong Wang equally contributed to this work.

Contents

Figure S1. *The asymmetry unit in **1***.. .…………........ . ...……… 2

Figure S2. *The coordination configurations for Y1, Y2 and Y3 ions in **1***.. ……....................……..2

Figure S3. *(**a-c**) The {Y1}$_4$ or {O}$_4$ constitutes a tetrahedron in **1**; (**d-g**) The {Y2-Y3}$_4$ or {O}$_4$ constitutes a tetrahedron in **1*** .. ……………………………………………………………….…2

Figure S4. *(**a-d**) View of the 3D simplified schematic diagram of **1** and the 3D framework with 1D open channels running in the [0 0 1] direction*…………………………………………………...3

Figure S5. *(**a**) View one of the nba^{2-} ligand connected with two B and one A clusters in **1**; (**b**) The simplied ligand 3-connected node (sym-metry codes: v, vi, viii, and ix see in the Table S3; xiv 1-x, 2-y, -1+z; xv 1-x, 1-y, -1+z).* ……………………………………………………...…..…….3

Figure S6. *(**a**) View one of the nba^{2-} ligand connected with two A and one B clusters in **1**; (**b**) The simplied ligand 3-connected node (symmetry codes: i, ii, iii, v, vi, and ix see in the Table S2; xv 1-x, 1-y,-1+z)* ……………………….………………………..…………………..…..……4

Figure S7. *Fluorescence intensity of 1 in different pH aqueous solution with the unintroduction ions (red) and introduction of F$^-$ (magenta)* ……………………………..…………...…..……4

Figure S8. *TG curves of compound **1***………………………………..………………..…..……5

Figure S9. *The infrared spectra of H$_2$nba ligand, **1** before and after F$^-$ sensing*..………..…..……5

Table S1. *Crystal structure data for **1**^{a,b,c}*….. ……………………………..………..…..……....6

Table S2. *Selected bond lengths/Å and bond angles/º for compound **1*** ………………………..…..7

Table S3. *Selected bond lengths (Å) and angles (deg) for **1** with estimated standard deviations in parentheses.* ……..……………………………………………......……………………..……..9

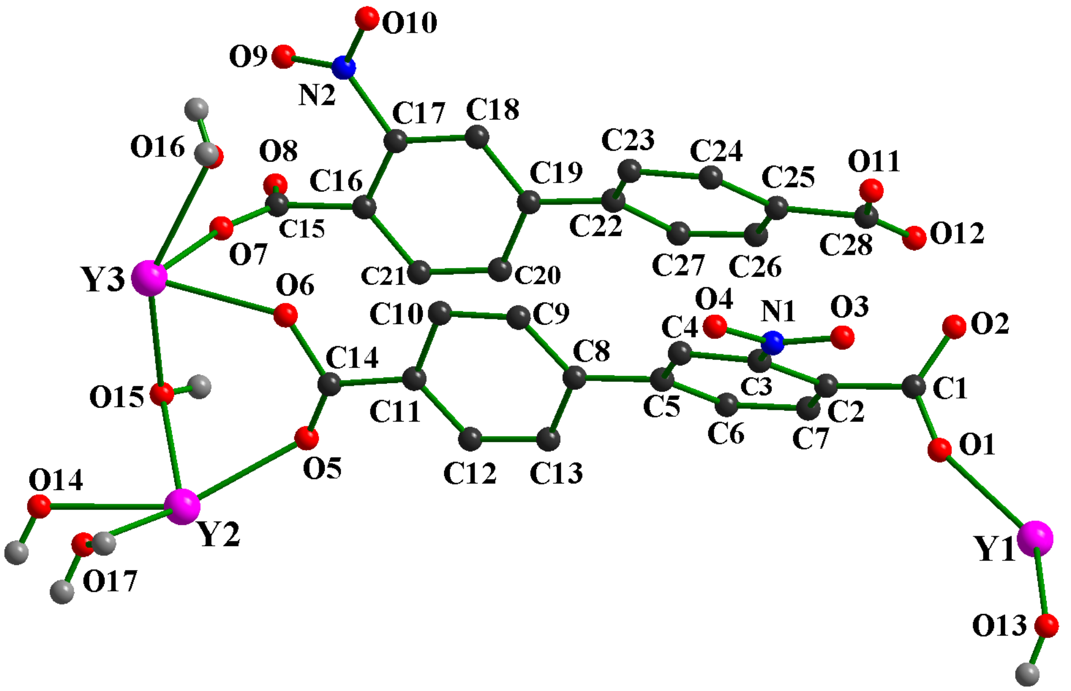

Figure S1. The asymmetry unit in **1**.

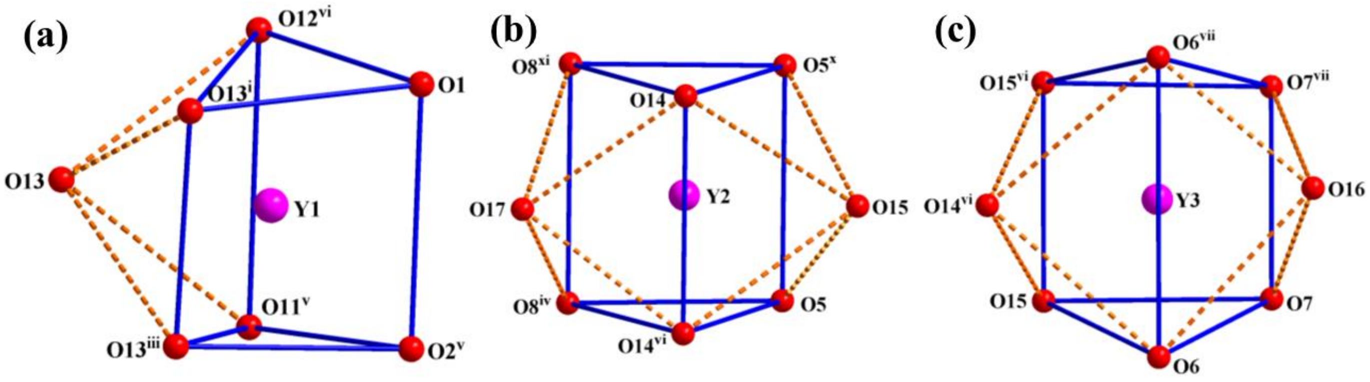

Figure S2. The coordination configurations for Y1, Y2 and Y3 ions in **1**.

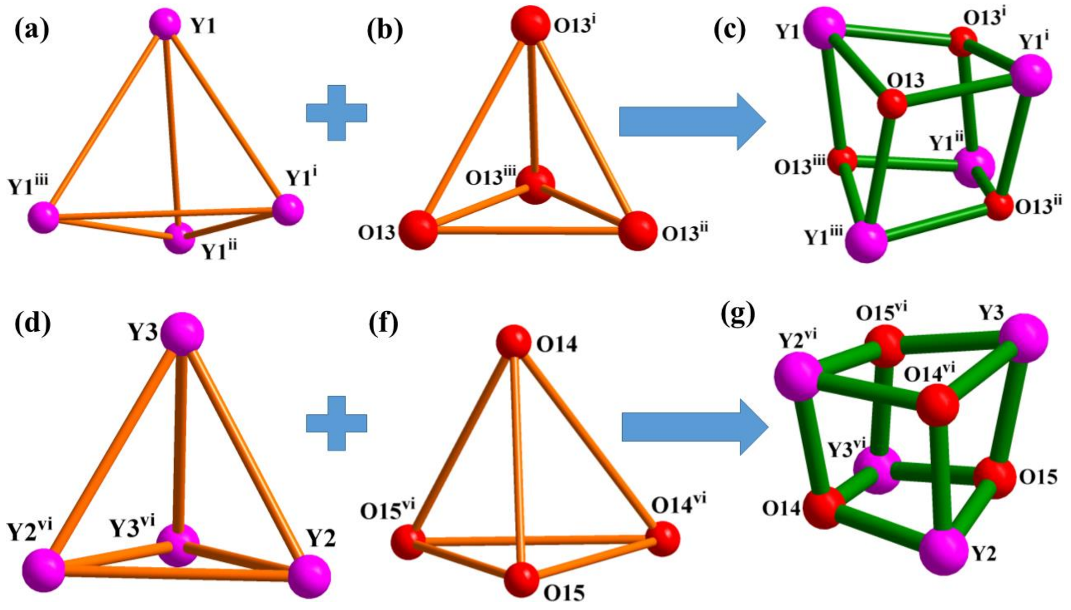

Figure S3. (**a-c**) The $\{Y1\}_4$ or $\{O\}_4$ constitutes a tetrahedron in **1**; (**d-g**) The $\{Y2\text{-}Y3\}_4$ or $\{O\}_4$ constitutes a tetrahedron in **1**.

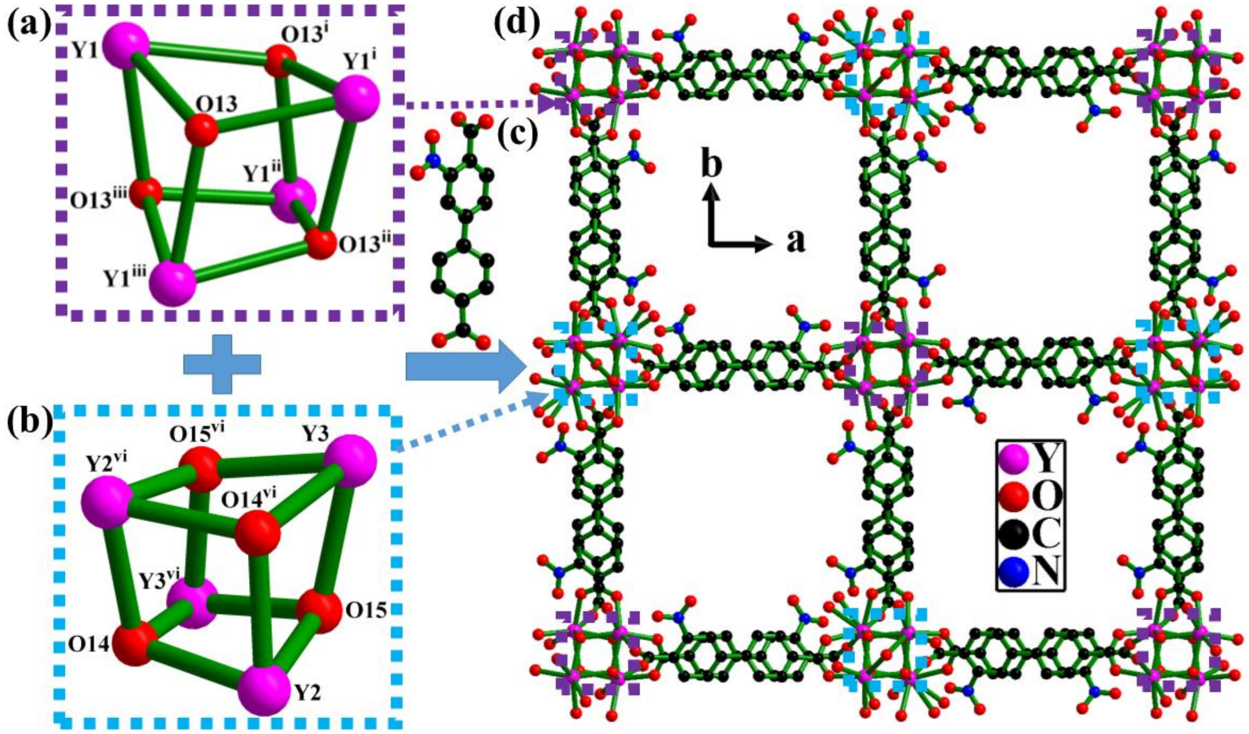

Figure S4. (**a-d**) View of the 3D simplified schematic diagram of **1** and the 3D framework with 1D open channels running in the [0 0 1] direction.

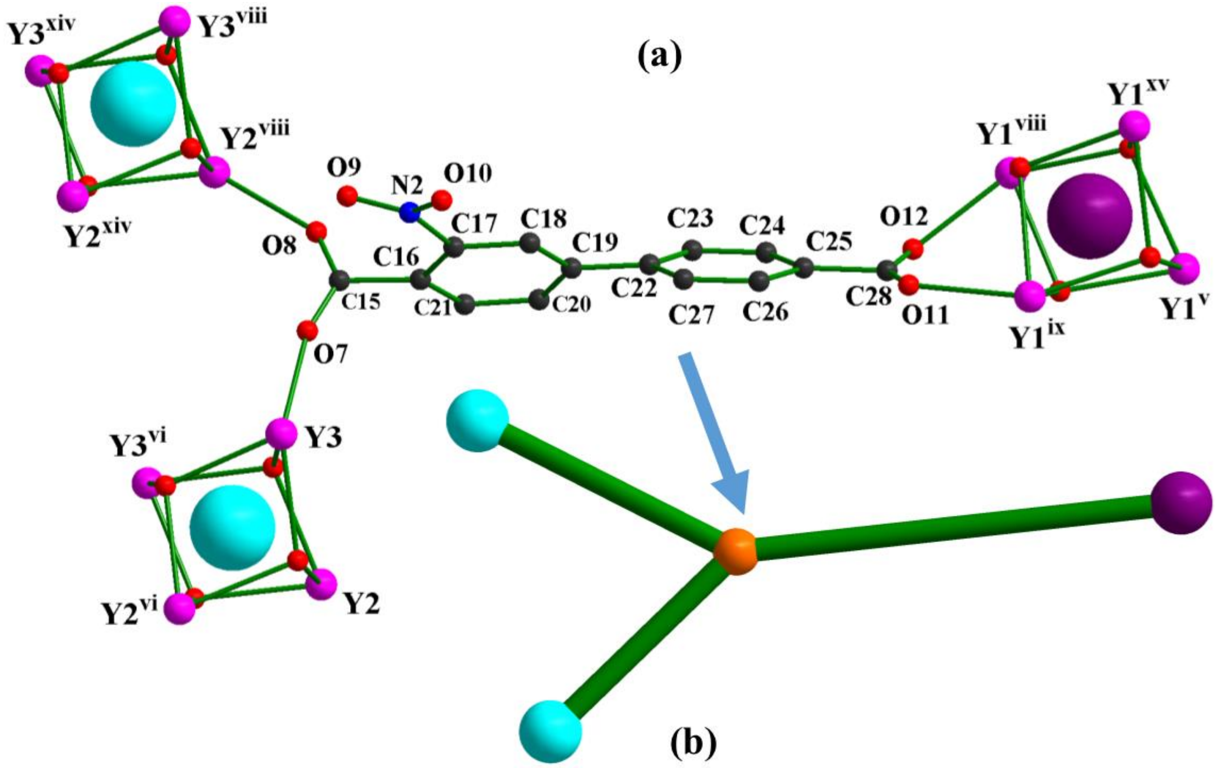

Figure S5. (**a**) View one of the nba^{2-} ligand connected with two B and one A clusters in **1**; (**b**) The simplied ligand 3-connected node (symmetry codes: v, vi, viii, and ix see in the Table S3; xiv 1-*x*, 2-*y*, -1+*z*; xv 1-*x*, 1-*y*, -1+*z*).

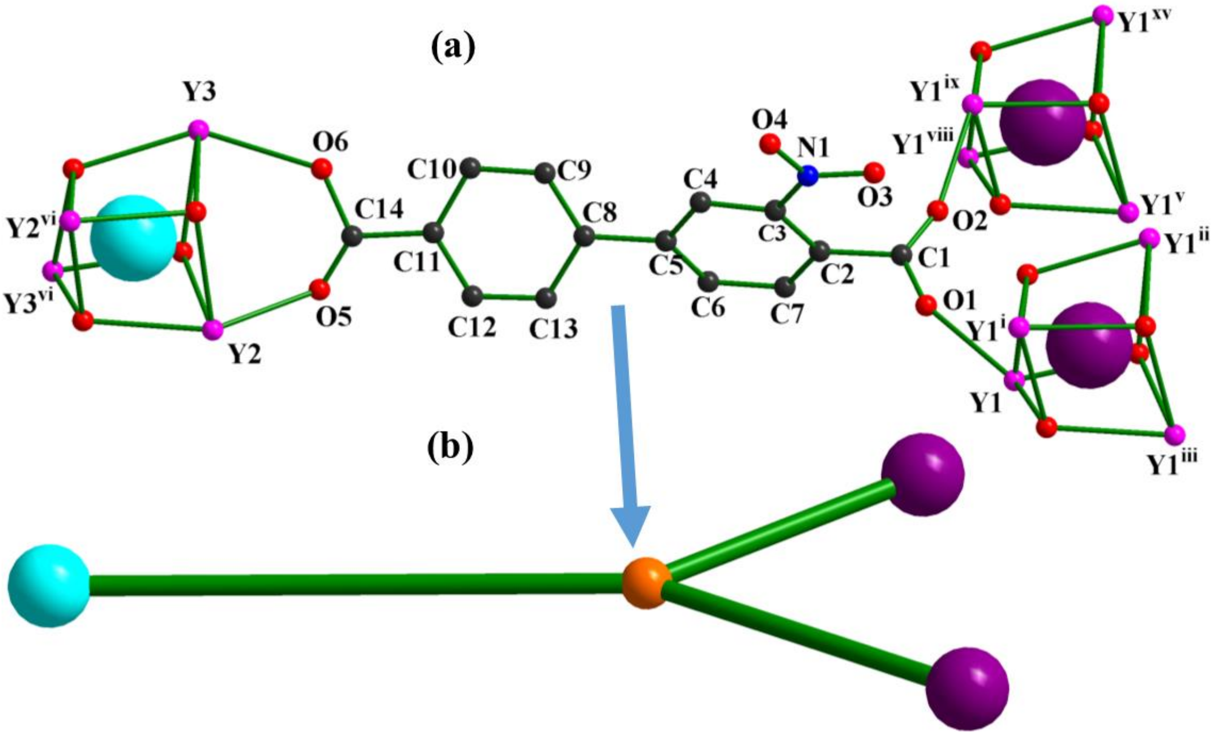

Figure S6. (**a**) View one of the nba^{2-} ligand connected with two A and one B clusters in **1**; (**b**) The simplied ligand 3-connected node (symmetry codes: i, ii, iii, v, vi, and ix see in the Table S2; xv 1-*x*, 1-*y*, -1+*z*).

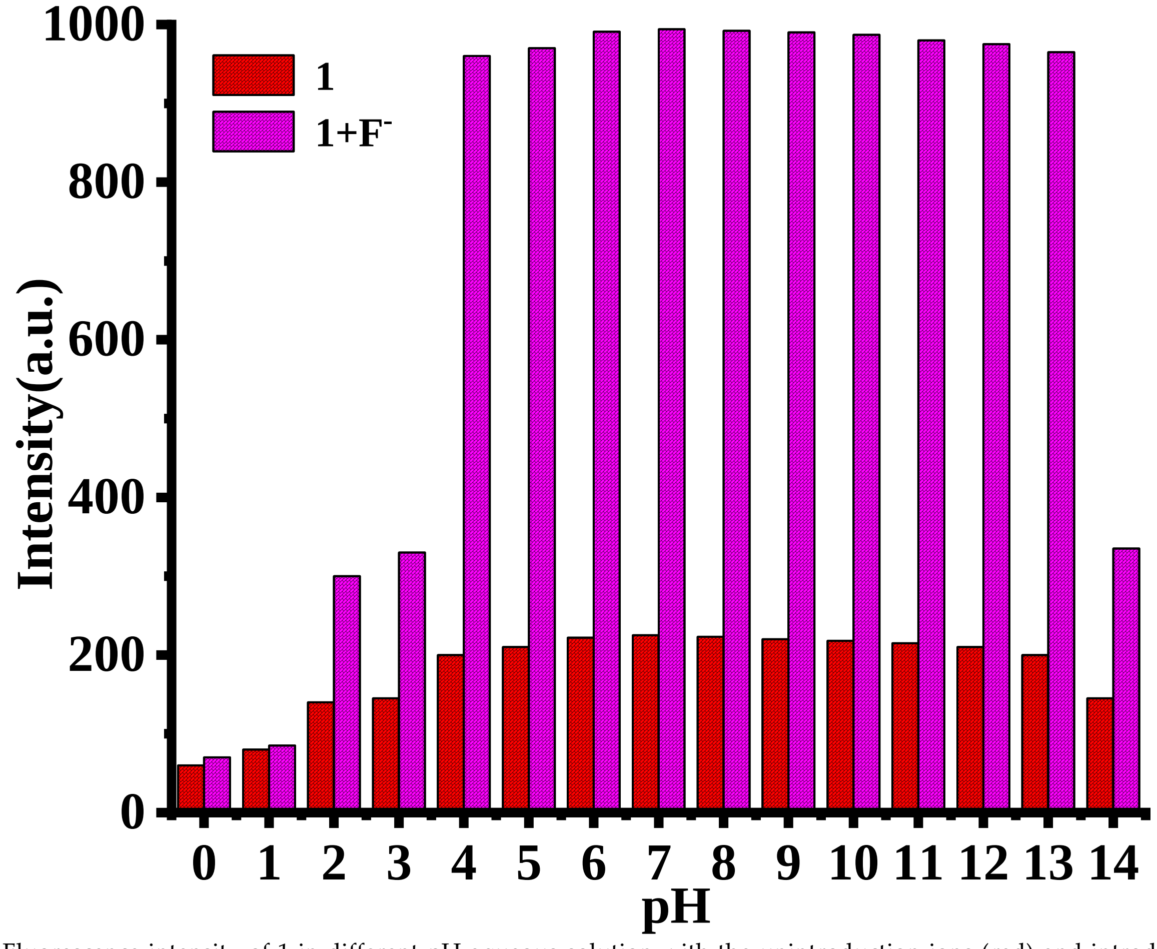

Figure S7. Fluorescence intensity of 1 in different pH aqueous solution with the unintroduction ions (red) and introduction of F$^-$ (magenta).

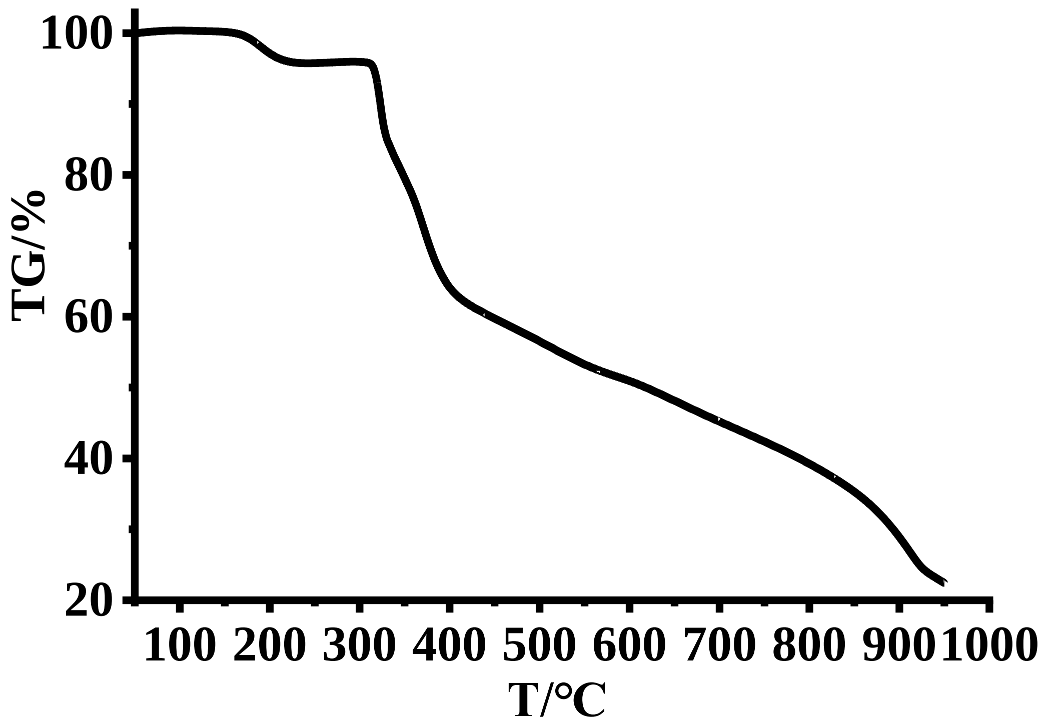

Figure S8. TG curves of compound **1**.

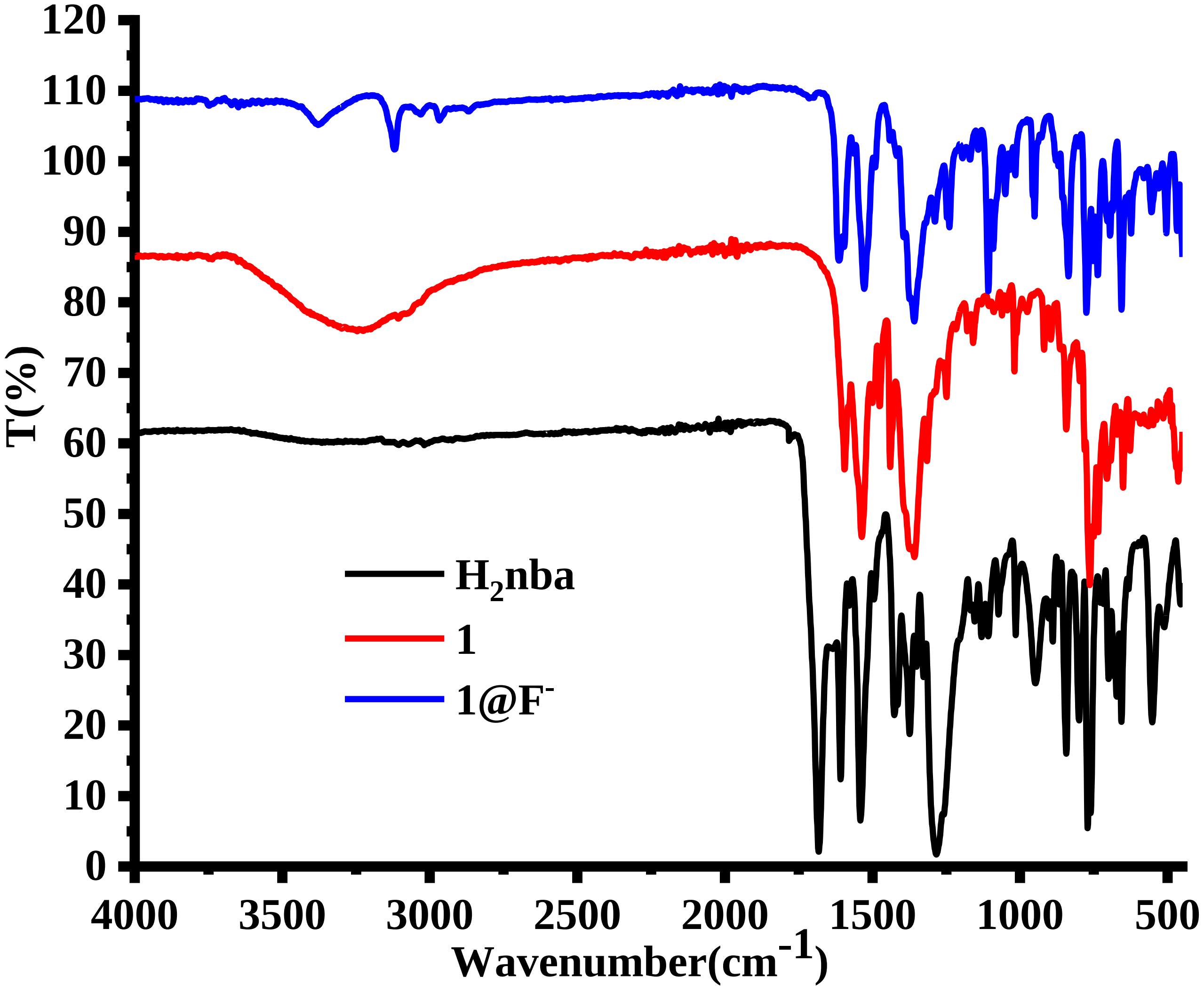

Figure S9. The infrared spectra of H_2nba ligand, **1** before and after F^- sensing.

Table S1. Crystal structure data for **1**[a,b,c].

Compound	**1**
Empirical formula	$C_{112}H_{70}N_8O_{59}Y_8$
Formula weight	3183.04
Crystal system	tetragonal
Space group	$P\bar{4}2_1m$
a, Å	33.7993(10)
b, Å	33.7993(10)
c, Å	7.9398(5)
α, deg	90
β, deg	90
γ, deg	90
V, Å^3	9070.4(8)
Z	2
D_{calcd}, g cm^{-3}	1.165
μ(MoK_α), mm^{-1}	2.598
F(000), e	3164
hkl range	-40→33, ±40, -7→9
$((\sin\theta)/\lambda)_{max}$, Å^{-1}	0.617
Refl. measured	33572
Refl. unique	8216
R_{int}	0.1128
Param. refined	178
$R_1{}^a$ / $wR_2{}^b$ [$I > 2\sigma(I)$]	0.1209/ 0.3069
$R_1{}^a$ / $wR_2{}^b$ (all data)	0.1419/0.3291
GoF (F^2)^c	1.053
$\Delta\rho_{fin}$ (max/min), e Å^{-3}	3.59/-4.68

[a] $R = \Sigma|| F_o| - |F_c|| / \Sigma|F_o|$; [b] $wR = [\Sigma w(F_o^2 - F_c^2)^2 / \Sigma w(F_o^2)^2]^{1/2}$, $w = [\sigma^2(F_o)^2 + (AP)^2 + BP]^{-1}$, where $P = (\mathrm{Max}(F_o^2,0)+2F_c^2)/3$; [c] GoF = $[\Sigma w(F_o^2 - F_c^2)^2/(n_{obs} - n_{param})]^{1/2}$.

Table S2. Selected bond lengths/Å and bond angles/º for compound **1**

Compound **1**[a]			
Y1-Y1^{i}	3.708(2)	Y2-Y2^{vi}	3.618(5)
Y1-Y1^{ii}	3.790(3)	Y3-Y3^{vi}	3.896(4)
Y1-Y1^{iii}	3.708(2)	Y3-Y2	3.761(3)
Y1-O1	2.280(14)	Y3-Y2^{vi}	3.761(3)
Y1-O11^{v}	2.333(15)	Y1-O2^{v}	2.344(15)
Y1-O13	2.463(16)	Y1-O13^{i}	2.353(14)
Y1-O13^{iii}	2.325(16)	Y1-O12^{iv}	2.315(15)
Y2-O5	2.320(15)	Y2-O5^{x}	2.309(15)
Y2-O8^{xi}	2.327(15)	Y2^{viii}-O8	2.333(15)
Y2-O14	2.378(14)	Y2-O14^{vi}	2.378(14)
Y2-O15	2.35(2)	Y2-O17	2.51(2)
Y3-O6	2.375(14)	Y3-O6^{vii}	2.375(15)
Y3-O7	2.264(16)	Y3-O7^{vii}	2.264(16)
Y3-O14^{vi}	2.37(2)	Y3-O15	2.412(13)
Y3-O16	2.49(2)	Y3-O15^{vi}	2.412(13)
O1-Y1-O12^{iv}	71.1(6)	O1-Y1-O11^{v}	131.5(6)
O1-Y1-O2^{v}	80.5(6)	O1-Y1-O13	135.8(6)
O1-Y1-O13^{i}	126.4(5)	O1-Y1-O13^{iii}	77.8(6)
O12^{iv}-Y1-O11^{v}	95.4(6)	O12^{iv}-Y1-O2^{v}	140.0(6)
O12^{iv}-Y1-O13^{iii}	89.5(6)	O12^{iv}-Y1-O13^{i}	147.7(6)
O12^{iv}-Y1-O13	73.9(5)	O11^{v}-Y1-O2^{v}	82.5(5)
O11^{v}-Y1-O13^{ii}	150.3(6)	O11^{v}-Y1-O13	77.5(5)
O2^{v}-Y1-O13	142.5(5)	O2^{v}-Y1-O13^{iii}	111.8(5)
O13^{i}-Y1-O11^{v}	90.0(5)	O13^{i} -Y1-O2^{v}	72.3(6)
O13^{i} -Y1-O13	76.3(6)	O13^{i}-Y1-O13^{iii}	71.2(6)
O13^{iii}-Y1-O13	75.8(6)	O6^{vi}-Y3-O6	105.8(8)
O6-Y3-O16	72.8(8)	O6^{vi}-Y3-O16	63.2(8)

O6-Y3-O15	83.6(6)	O6-Y3-$O15^{vii}$	143.2(7)
$O6^{vi}$-Y3-O15	143.2(7)	$O6^{vi}$-Y3-$O15^{vii}$	83.6(6)
$O5^{x}$-Y2-O5	80.8(9)	O5-Y2-$O8^{xi}$	122.9(6)
O5-Y2-$O8^{iv}$	72.2(5)	O7-Y3-O6	79.8(6)
$O5^{x}$-Y2-$O8^{iv}$	122.9(6)	$O7^{vi}$-Y3-O6	140.1(6)
$O5^{x}$-Y2-$O8^{xi}$	72.3(5)	$O7^{vi}$-Y3-$O6^{vi}$	79.8(6)
O7-Y3-$O6^{vi}$	140.1(6)	O7-Y3-$O7^{vi}$	72.5(8)
$O5^{x}$-Y2-$O14^{vii}$	145.0(7)	O7-Y3-O16	82.0(8)
O5-Y2-O14	145.0(7)	$O7^{vi}$-Y3-O16	75.3(7)
O5-Y2-$O14^{vii}$	89.8(6)	$O5^{x}$-Y2-O14	89.8(6)
$O5^{x}$-Y2-O17	138.9(4)	O5-Y2-O17	138.9(4)
O5-Y2-O15	71.1(6)	$O7^{vi}$-Y3-$O14^{vii}$	142.0(4)
$O5^{x}$-Y2-O15	71.1(6)	O7-Y3-$O14^{vii}$	142.0(4)
O7-Y3-O15	76.1(7)	$O7^{vi}$-Y3-$O15^{vii}$	76.1(7)
O7-Y3-$O15^{vii}$	115.8(6)	$O7^{vi}$-Y3-O15	115.8(6)
$O8^{xi}$-Y2-$O8^{iv}$	81.6(8)	$O8^{iv}$-Y2-O14	138.0(6)
$O8^{iv}$-Y2-$O14^{vii}$	85.1(7)	$O8^{xi}$-Y2-O14	85.1(6)
$O8^{xi}$-Y2-$O14^{vii}$	138.0(6)	$O8^{xi}$-Y2-O17	75.0(6)
$O14^{vii}$-Y3-$O6^{vi}$	75.3(5)	$O8^{iv}$-Y2-O17	75.0(6)
$O14^{vii}$-Y3-O6	75.3(5)	$O8^{iv}$-Y2-O15	137.4(4)
$O14^{vii}$-Y3-O16	116.4(8)	$O14^{vii}$-Y3-O15	73.0(7)
$O8^{xi}$-Y2-O15	137.4(4)	$O14^{vii}$-Y3-$O15^{vii}$	73.0(7)
$O15^{vii}$-Y3-O16	139.2(9)	O15-Y3-O16	150.3(9)
$O14^{vii}$-Y2-O14	78.7(9)	$O14^{vii}$-Y2-O17	63.2(6)
O14-Y2-O17	63.2(6)	$O15^{vii}$-Y3-O15	69.7(9)
O15-Y2-$O14^{vii}$	74.0(7)	O15-Y2-O14	74.0(7)

[a]Symmetry codes: i *y*, 1-*x*, 3-*z*; ii 1-*x*, 1-*y*, *z*; iii 1-*y*, *x*, 3-*z*; iv *x*, *y*, 1+*z*; v 1-*y*, *x*, 2-*z*; vi 1-*x*, 2-*y*, *z*; vii 3/2-*y*, 3/2-*x*, *z*; viii *x*, *y*, -1+*z*; ix *y*, 1-*x*, 2-*z*; x -1/2+*y*, 1/2+*x*, *z*; xi -1/2+*y*, 1/2+*x*, 1+*z*.

Table S3. Selected bond lengths (Å) and angles (deg) for **1** and **2** with estimated standard deviations in parentheses.

D-H···A	d(D-H)	d(H···A)	d(D...A)	∠(DHA)
Compound **1**[a]				
O17-H17A···O7^{vi}	0.85	2.17	2.88(2)	141.4
O17-H17A···O7^{xi}	0.85	2.17	2.88(2)	141.4
O17-H17B···O7^{xii}	0.85	2.17	2.88(2)	141.4
O17-H17B···O7^{xiii}	0.85	2.17	2.88(2)	141.4
O13-H13A···O2^{iv}	0.85	2.44	2.76(2)	102
O14-H14···O9^{xii}	0.85	2.49	3.25(2)	150
O14-H14···O9^{xi}	0.85	2.49	3.25(2)	150
O16-H16A···N2^{vii}	0.85	2.30	3.15(3)	174
C26 -H26···O12	0.93	2.39	2.74(2)	101

[a]Symmetry codes: iv *x*, *y*, 1+*z*; vi 1-*x*, 2-*y*, *z*; vii 3/2-*y*, 3/2-*x*, *z*; xi -1/2+*y*, 1/2+*x*, 1+*z*; xii 1-*x*, 2-*y*, 1+*z*; xiii 3/2-*y*, 3/2-*x*, 1+*z*.
